# Hsa_circ_0057105 modulates a balance of epithelial‐mesenchymal transition and ferroptosis vulnerability in renal cell carcinoma

**DOI:** 10.1002/ctm2.1339

**Published:** 2023-07-26

**Authors:** Junjie Cen, Yanping Liang, Zihao Feng, Xu Chen, Jinlong Chen, Yinghan Wang, Jiangquan Zhu, Quanhui Xu, Guannan Shu, Wenbin Zheng, Hui Liang, Zhu Wang, Qiong Deng, Jiazheng Cao, Junhang Luo, Xiaohan Jin, Yong Huang

**Affiliations:** ^1^ Department of Urology The First Affiliated Hospital of Sun Yat‐sen University Guangzhou People's Republic of China; ^2^ Department of Laboratory Medicine The First Affiliated Hospital of Sun Yat‐sen University Guangzhou People's Republic of China; ^3^ Department of Emergency The First Affiliated Hospital of Sun Yat‐sen University Guangzhou People's Republic of China; ^4^ Department of Urology Affiliated Longhua People's Hospital Southern Medical University Shenzhen People's Republic of China; ^5^ Department of Urology Jiangmen Central Hospital Jiangmen People's Republic of China; ^6^ Institute of Precision Medicine The First Affiliated Hospital of Sun Yat‐sen University Guangzhou People's Republic of China

**Keywords:** circRNA, EMT pathway, ferroptosis, renal cell carcinoma

## Abstract

**Background:**

The incidence of renal cell carcinoma (RCC) has increased in recent years. Metastatic RCC is common and remains a major cause of mortality. A regulatory role for circular RNAs (circRNAs) in the occurrence and progression of RCC has been identified, but their function, molecular mechanisms, and potential clinical applications remain poorly understood.

**Methods:**

High‐throughput RNA sequencing was used to explore the differential expression of circRNAs and their related pathways in RCC patients. Transwell and CCK‐8 assays were used to assess the function of hsa_circ_0057105 in RCC cells. The clinical relevance of hsa_circ_0057105 was evaluated in a cohort of RCC patients. The hsa_circ_0057105 regulatory axis was defined using RNA pull‐down, luciferase reporter assays, and fluorescence in situ hybridization assays, and the in vivo effect of hsa_circ_0057105 was validated using animal experiments.

**Results:**

Single‐sample gene set enrichment analysis and correlation analysis of RNA‐seq data showed that hsa_circ_0057105 was potentially oncogenic and may serve to regulate epithelial‐mesenchymal transition (EMT) activation in RCC. Hsa_circ_0057105 expression was associated with advanced TNM stages and was an independent prognostic factor for poor RCC patient survival. Phenotypic studies show that hsa_circ_0057105 can enhance the migration and invasion abilities of RCC cells. Further, hsa_circ_0057105 was shown to inhibit the expression of miR‐577, a miRNA that regulated the expression of both COL1A1, which induced EMT activation, and VDAC2, which modulated ferroptosis sensitivity. The dual regulatory roles of hsa_circ_0057105 on EMT and ferroptosis sensitivity were verified using rescue experiments. Animal studies confirmed that hsa_circ_0057105 increased the metastatic ability and ferroptosis sensitivity of RCC cells in vivo.

**Conclusions:**

In RCC, hsa_circ_0057105 regulates COL1A1 and VDAC2 expression through its sponge effect on miR‐577, acting like a ‘double‐edged sword’. These findings provide new insight into the relationship between EMT and ferroptosis in RCC and provide potential biomarkers for RCC surveillance and treatment.

## Graphical abstract

In renal cell carcinoma (RCC), hsa_circ_0057105 acts like a 'double‐edged sword’. On one hand, it promotes the metastasis of RCC cells through activation of epithelial‐mesenchymal transition. On the other hand, it also increases the ferroptosis sensitivity of RCC cells. This suggests that under the modulation of hsa_circ_0057105, RCC cells need to choose between ‘aggressiveness’ and ‘oxidative vulnerability’.

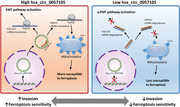

## 1 INTRODUCTION

The incidence of renal cell carcinoma (RCC) has increased in recent years and now accounts for about 3% of all cancers.^1^ RCC is the most common cancer of renal origin, responsible for approximately 90% of all kidney‐specific malignancies,^2^ and many cases are discovered incidentally during abdominal imaging for other conditions.^3^ The most common RCC subtype, accounting for 80% of cases, is clear cell renal cell carcinoma (ccRCC).^4^ In clinical setting, even following systemic treatment with sunitinib, the median recurrent‐free survival of metastatic RCC is approximately 12 months.^5^ Characterizing the mechanism of RCC metastasis is vital for disease monitoring and treatment development, however, the current understanding of RCC metastasis remains limited.

Circular RNAs (circRNAs) belong to a subclass of non‐coding RNAs. CircRNAs are produced through a non‐canonical splicing process known as back‐splicing.^6^ During this process, the 5′ and 3′ ends of a linear mRNA are linked together to form a circular structure. CircRNAs were previously defined as the ‘junk’ of transcription.^7^ However, many functional circRNAs have been discovered using high‐throughput RNA sequencing (RNA‐seq) and circRNA‐identifying bioinformatic algorithms.^8^ Many of these circRNAs exert tumour‐specific functions and help to regulate cancer progression and metastasis.^9^ Our previous study identified the circSDHC axis in RCC and showed that it regulates RCC proliferation and metastasis.^10^ However, the function of circRNA in RCC remains largely elusive, and more studies are needed to develop additional treatment options.

Epithelial‐mesenchymal transition (EMT) is a reversible cellular process that occurs during embryogenesis, wound healing, and cancer progression.^11^ Epithelial cells develop a spindle‐shaped, mesenchymal morphology and acquire increased mobility during EMT process.^12^ EMT plays an indispensable role in the progression and metastasis of many cancers, including breast,^13^ lung,^14^ colorectal,^15^ and renal cancer.^16^ Recent studies have also shown that EMT is closely linked to ferroptosis of tumour cells.^17^ Ferroptosis is a type of non‐apoptotic programmed cell death that is characterized by the accumulation of reactive oxygen species and lipid peroxidation products in an iron‐dependent manner.^18^ Interestingly, while EMT makes tumour cells more aggressive, the adoption of a mesenchymal state increases cell sensitivity to ferroptosis, which has been already reported in head and neck cancer,^19^ lung cancer,^20^ and adrenal adenocarcinoma.^21^ However, the relationship between EMT and ferroptosis has not yet been reported or characterized in RCC.

The current study identified a circRNA, hsa_circ_0057105, that regulated COL1A1 and VDAC2 expression through the sponge effect on miR‐577 and created a balance between EMT and ferroptosis sensitivity in RCC cells. On one hand, it promotes the metastasis of RCC cells through activation of EMT. On the other hand, it also increases the ferroptosis sensitivity of RCC cells. These findings provide new insight into the relationship between EMT and ferroptosis in RCC and provide potential biomarkers for RCC surveillance and treatment.

## 2 MATERIALS AND METHODS

### 2.1 Clinical samples and patient follow‐up data

The collection and use of all patient samples and clinical data were approved by the Ethical Committees of the First Affiliated Hospital of Sun Yat‐sen University (Guangzhou, China). Informed consent was obtained from the patients. Five pairs of fresh ccRCC tumour and normal adjacent parenchyma tissue were collected during surgery and mRNA plus circRNA sequencing were conducted. Patient samples and clinical data from 130 ccRCC patients who underwent surgery in our institution from January 2002 to December 2012 were collected. The median follow‐up time was 102 months. Median hsa_circ_0057105 expression was used as a cut‐off point in chi‐square analysis and Kaplan–Meier survival analysis. Overall survival (OS) was defined as the duration from the date of surgery to the date of patient's death for any reason. Recurrence‐free survival (RFS) was defined as the duration from the date of surgery to the date of first notice of disease recurrence. Total RNA was extracted from formalin‐fixed, paraffin‐embedded (FFPE) samples using an RNA isolation kit for FFPE (ThermoFisher, MA, USA). The kidney renal clear cell carcinoma (KIRC/ccRCC) dataset from The Cancer Genome Atlas (TCGA) was retrieved from the cBioPortal website (https://www.cbioportal.org/).

### 2.2 Bioinformatics

Differential expression analysis of the mRNAs and circRNAs was conducted with R software v4.1.3 (https://www.r‐project.org/) using the tidyverse, limma and pheatmap packages. CircBase (http://circbase.org/) was used to annotate the differential circRNAs. GEPIA (http://gepia.cancer‐pku.cn/) was used to query the survival analysis associated with particular genes. Gene set enrichment analysis (GSEA, https://www.gsea‐msigdb.org/gsea) was conducted to compare the activated pathways between different patient groups. GSEA network map was generated with Cytoscape 3.9.1 (https://cytoscape.org/). Single‐sample gene set enrichment analysis (ssGSEA, https://www.genepattern.org/) was conducted to assess the activation status of particular pathways in each sample. CircPrimer2.0 software^22^ was used to design and validate the circRNA primers. To predict potential‐circRNA interactions, two databases, ENCORI (https://starbase.sysu.edu.cn/) and Circular RNA Interactome (https://circinteractome.nia.nih.gov/index.html) were queried. Two groups of predictions were found to overlap, yielding interactions with a positive predictive value.

### 2.3 Cell lines and culture

Two normal kidney cell lines (HK2 and 293) and four RCC cell lines (Caki‐1, A498,769P, and 786‐O) were obtained from American Type Culture Collection (ATCC). HK2, 769P, and 786‐O cells were cultured in RPMI 1640 medium (Gibco, NY, USA) with 10% fetal bovine serum (FBS, ExCell Bio, Shanghai, China) while 293, A498, and Caki‐1 cells were cultured in DMEM medium (Gibco) with 10% FBS (ExCell Bio). All cells were maintained in a bio‐incubator (ThermoFisher) adjusted to 37°C and 5% CO_2_. Mycoplasma infection was periodically checked using a detcction Kit (Beyotime, Shanghai, China).

### 2.4 Animal experiments

The animal experiments performed in this study were reviewed and approved by the Institutional Animal Care and Use Committee of Sun Yat‐sen University. Three‐week‐old nude BALB/c mice were purchased from GemPharmatech (Guangdong, China). Caki‐1 cells were stably transfected with the control or hsa_circ_0057105 overexpression vector. For the subcutaneous tumour formation model, 20 mice were randomly divided into four groups, two groups receiving Caki‐1 (the control vector) and two receiving Caki‐1 (hsa_circ_0057105 overexpression). Approximately 5 × 10^6^ cells were inoculated subcutaneously into the right side of each mouse. After 2 weeks of inoculation, erastin treatment was initiated (15 mg/kg intraperitoneal, twice every other day). Corn oil (Solarbio, Beijing, China) supplemented with 5% DMSO was used as a solvent for the erastin. The total treatment time was 30 days and tumour volume was measured every week. For the tail‐vein metastasis model, 16 mice were randomly distributed into two groups, one group receiving Caki‐1 (control vector) and one receiving Caki‐1 (hsa_circ_0057105 overexpression). Approximately 2 × 10^6^ cells were injected into each mouse intravenously via the tail vein. Luciferase activity near the pulmonary area was measured every 2 weeks using an IVIS Spectrum In Vivo Imaging System (PerkinElmer, MA, USA). The mice were euthanized 8 weeks after injection and the lungs were collected, fixed, and HE stained. The number of metastatic foci was determined using a BX63F Upright Microscope (Olympus), and representative images were captured. For human tumour cells detection in the mouse circulation, before the euthanization, mice were anesthetized and perfused with 5 ml of PBS via the left ventricle. From the right atrium, 3 ml of blood was collected. Then, the blood was treated with ACK lysis buffer (ThermoFisher) and RNA was extracted. Quantitative real‐time PCR (qRT‐PCR) was used to detect the human GAPDH level. Murine β2‐microglobulin was used as an internal control.

### 2.5 Statistical analysis

GraphPad Prism 9 was utilized to conduct statistical analyses and plot graphs. Comparisons between two groups were conducted using the student's t‐test. Comparisons among three or more groups were determined using the analysis of variance. Correlation between continuous variables was determined using Pearson's analysis. Association analysis between low/high hsa_circ_0057105 patient groups and clinical parameters was performed using Chi‐square analysis. Survival analysis was shown using Kaplan–Meier curves and significance was determined using the log‐rank test. Univariate and multivariate analyses of OS and RFS were conducted using Cox regression. All experiments were performed three times. Quantitative data are presented as the mean ± standard deviation. All *p* values < 0.05 were considered significant (*p* < 0.05 for *; *p* < 0.01 for **; *p* < 0.001 for ***). For other detailed experiment protocols, please refer to Supplemental Materials and methods.

## 3 RESULTS

### 3.1 Identification of EMT‐related circRNAs using high‐throughput sequencing

**FIGURE 1:**
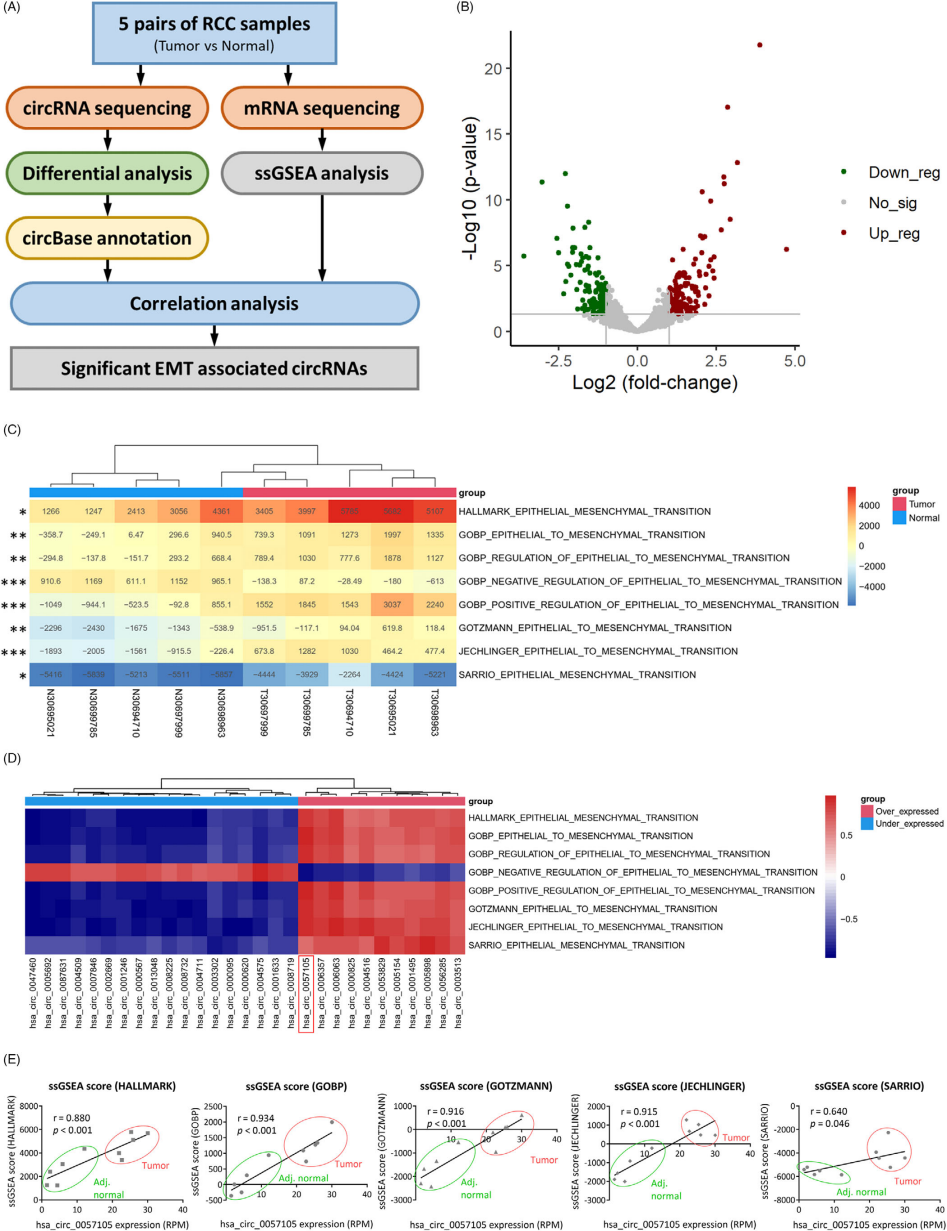
Identification of epithelial‐mesenchymal transition (EMT)‐related circRNAs using high‐throughput sequencing. (A) Flowchart of high‐throughput screening and analysis steps of five pairs of renal cell carcinoma (RCC) samples from our institute. (B) Volcano plot of significant circRNAs in the sequencing dataset (>2‐fold change, *p* < 0.05). Red dots, green dots and grey dots indicate up‐regulated, down‐regulated and not significant circRNAs, respectively. (C) Heatmap of 8 EMT‐related pathway single‐sample gene set enrichment analysis (ssGSEA) scores in five pairs of RCC samples. Asterisks on the left indicate the significance of difference between tumour and normal adjacent samples. Student's *t*‐test is applied. (D) Heatmap of significant correlation coefficients from all circRNAs expression and EMT‐related pathway ssGSEA scores. (E) Separated correlation analysis of hsa_circ_0057105 expression and 5 classic EMT‐related pathway ssGSEA scores. Red circles and green circles indicate tumour and normal adjacent sample (adj. normal) datapoints, respectively.

To comprehensively analyze circRNAs and their related pathways in RCC, five matched fresh ccRCC and normal adjacent kidney samples were collected during surgery and subjected to circRNA and mRNA sequencing (Figure 1A). For circRNA‐seq analysis, differentially expressed circRNAs (>2‐fold change, *p* < 0.05) with circBase annotation were selected, yielding 212 significant circRNAs (Figure 1B and Table S1). The top 20 over/under‐expressed circRNAs were shown in a heatmap (Figure S1A). For pathway analysis, single‐sample gene set enrichment analysis (ssGSEA) was performed using mRNA‐seq as the input. The ssGSEA scores of three RCC tumourigenesis‐related pathways were significantly differ between tumour and normal adjacent kidney samples (Figure S1B), indicating the validity of the sequencing result. Epithelial‐mesenchymal transition (EMT) activation, an important event associated with RCC progression, was shown to differ significantly between tumour and normal adjacent kidney samples (Figure 1C and Figure S1C). To determine if any of the differentially expressed circRNAs had the potential to regulate EMT activation and further promote RCC progression, a comprehensive correlation analysis between differentially expressed circRNAs and EMT ssGSEA scores was performed, and a heatmap including all the significant correlations was constructed (Figure 1D and Table S2). Of the pro‐EMT circRNAs, hsa_circ_0057105 was the most significantly associated with EMT ssGSEA scores, showing correlation coefficients more than 0.8 with several classic EMT gene sets (Figure 1E). In the mRNA‐seq data, hsa_circ_0057105 expression had a negative correlation with CDH1 (E‐cadherin, E‐cad) and positive correlations with CDH2 (N‐cadherin, N‐cad) and Vimentin (VIM) expression, consistent with the EMT activation pattern (Figure S1D).

### 3.2 Characterization of hsa_circ_0057105 in RCC

**FIGURE 2:**
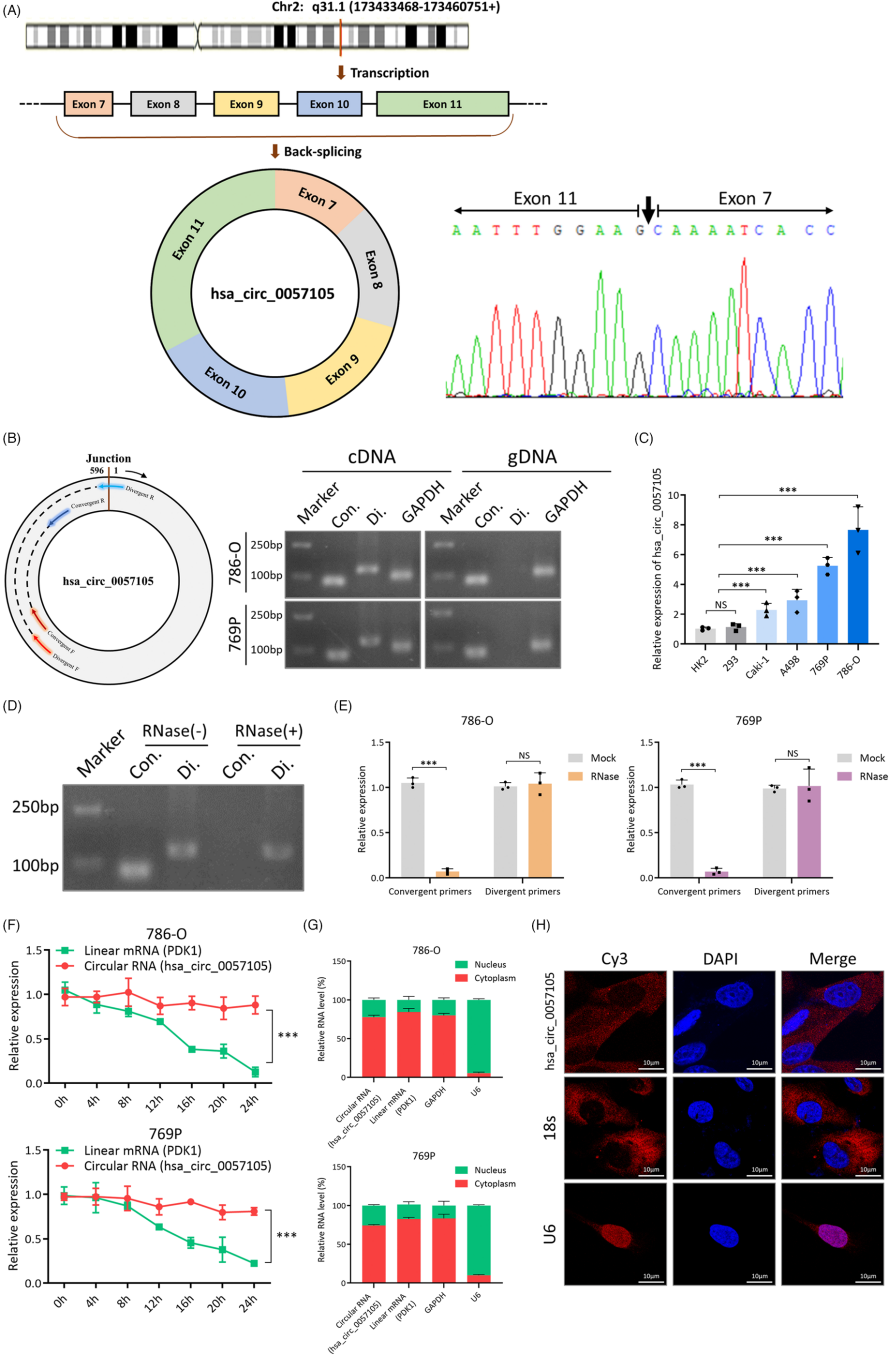
Characterization of hsa_circ_0057105 in renal cell carcinoma (RCC). (A) (Left) Chromosomal origin of hsa_circ_0057105 and its formation via back‐splicing. (Right) Sanger sequencing confirmation of hsa_circ_0057105 back‐splicing junction, indicated by black arrow. (B) (Left) Design of convergent and divergent primers of within hsa_circ_0057105 circle. (Right) DNA electrophoresis of PCR products amplified from 786‐O and 769P cells. Con.‐convergent primers. Di.‐divergent primers. GAPDH was used as positive control. (C) Expression of hsa_circ_0057105 in normal kidney cell line (HK2 and 293) and four RCC cell lines (A498, Caki‐1, 769P and 786‐O). (D) DNA electrophoresis of PCR products amplified from 786‐O with/without RNase R treatment. Con.‐convergent primers. Di.‐divergent primers. (E) qRT‐PCR detection of linear mRNA and circular hsa_circ_0057105 in 786‐O (left) and 769P (right) cells. Expression levels were normalized to the mock group. (F) Actinomycin D assays of linear mRNA and circular hsa_circ_0057105 in 786‐O (upper) and 769P (lower) cells. RNA levels were determined by qRT‐PCR. Expression levels were normalized to 0 h. (G) Detection of linear mRNA and circular hsa_circ_0057105 in nuclear/cytoplasmic fractions isolated from 786‐O (upper) and 769P (lower) cells. RNA levels were determined by qRT‐PCR. (H) FISH experiment detecting the subcellular localization of hsa_circ_0057105 in 786‐O cells. 18s was used as positive cytoplasm control. U6 was used as positive nucleus control. Nuclei were stained with DAPI (4',6‐diamidino‐2‐phenylindole). Data are presented as mean ± SD, *n* = 3.

Hsa_circ_0057105 is derived from the PDK1 gene on chromosome 2. After transcription, the pre‐mRNA of PDK1 undergoes back‐splicing and results in the circulation of exon 7 to exon 11 (Figure 2A). With a primer that specifically targeted the back‐splicing site, Sanger sequencing was able to identify hsa_circ_0057105 (Figure 2A). Using convergent/divergent primers, hsa_circ_0057105 was amplified from cDNA but not gDNA, meaning that it was formed as a result of posttranscriptional back‐splicing (Figure 2B). Four RCC cell lines, Caki‐1, A498, 769P, and 786‐O, demonstrated higher expression of hsa_circ_0057105 than the normal kidney cell lines, HK2 and 293, indicating that it is likely to have an oncogenic function (Figure 2C). Compared to its linear form, hsa_circ_0057105 also had a higher resistance to RNase digestion (Figure 2D,E) and a longer half‐life (Figure 2F). During nucleocytoplasmic fractionation, the majority of hsa_circ_0057105 localized to the cytoplasm, and this was later confirmed using fluorescence in situ hybridization (FISH) subcellular localization (Figure 2G,H).

### 3.3 Hsa_circ_0057105 is an oncogenic circRNA in RCC patients

**FIGURE 3:**
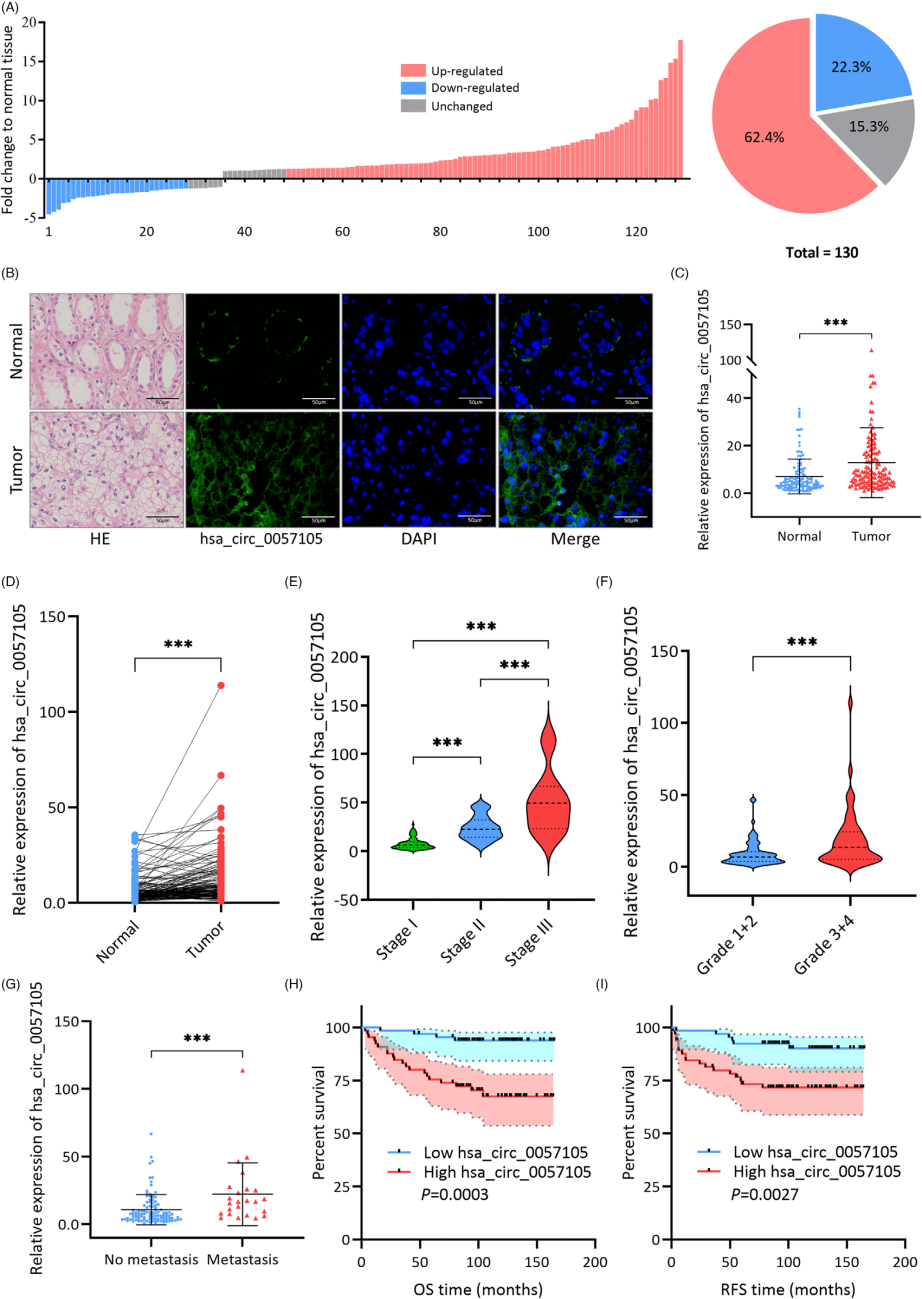
Hsa_circ_0057105 is an oncogenic circRNA in renal cell carcinoma (RCC) patients. (A) Summary of hsa_circ_0057105 expression in tumour compared to normal adjacent tissue from 130 ccRCC patients. RNA levels were determined by qRT‐PCR. GAPDH was used as an internal control. (B) HE staining showing the cellular structures and FISH experiment detecting the hsa_circ_0057105 in patient tumour and normal adjacent tissue samples. Hsa_circ_0057105 was stained with FAM (carboxyfluorescein). Nuclei were stained with DAPI. (C) Expression of hsa_circ_0057105 in patient tumour and normal adjacent tissue samples. (D) Paired analysis of hsa_circ_0057105 expression in patient tumour and normal adjacent tissue samples. (E) Expression of hsa_circ_0057105 in patient groups with different clinical stages (TNM, T: primary tumour, N: regional lymph nodes, M: distant metastasis). (F) Expression of hsa_circ_0057105 in patient groups with different Fuhrman grades. (G) Expression of hsa_circ_0057105 in patient groups who developed/not developed metastasis during follow‐up period. (H and I) Survival analysis of OS and RFS in patient groups with high and low hsa_circ_0057105 expression (median expression as cut‐off), presented as Kaplan–Meier curve. Significance was determined by Log‐rank test. RNA levels in (C–I) were determined by qRT‐PCR. GAPDH was used as an internal control. Data are presented as mean ± SD, *n* = 3.

To evaluate the clinical significance of hsa_circ_0057105 in RCC patients, 130 pairs of tumour and normal adjacent tissue samples were collected from ccRCC patients who underwent surgery in our hospital. In both the quantitative real‐time PCR (qRT‐PCR) and FISH experiments, tumours demonstrated significantly diffuse and higher expression of hsa_circ_0057105 than normal adjacent tissue, which was focal and confined in the proximal convoluted tubules (Figure 3A–D). Moreover, as the clinical stage and Fuhrman grade progressed, hsa_circ_0057105 expression increased substantially (Figure 3E,F). Higher expression of hsa_circ_0057105 was also detected in those patients who later developed metastasis during follow‐up (Figure 3G). Using a median expression of hsa_circ_0057105 as the cut‐off point in survival analysis, patients in the high expression group had lower overall survival (OS) and recurrence‐free survival (RFS) (Figure 3H,I). Chi‐square analysis showed that hsa_circ_0057105 was significantly associated with advanced clinical stage and higher Fuhrman grade (Table 1). Univariate and multivariate Cox regression analysis showed that hsa_circ_0057105 was an independent prognostic factor for both OS and RFS (OS univariate HR: 3.498, 95% CI: 1.636−7.477; OS multivariate HR: 3.028, 95% CI: 1.346−6.812; RFS univariate HR: 2.449, 95% CI: 1.273−4.710; RFS multivariate HR: 2.123, 95% CI: 1.038−4.340) (Tables 2 and 3).

**TABLE 1:** Association of hsa_circ_0057105 expression with clinicopathological characteristics in 130 ccRCC patients.

Parameter	Total	hsa_circ_0057105 expression		*p* Value
		High	Low	
**Age (y)**				
<60	95	46	49	0.693
≥60	35	19	16	
**Gender**				
Female	41	25	16	0.131
Male	89	40	49	
**Clinical (TNM) stage**				
I	101	38	63	<0.001
II	22	20	2	
III	7	7	0	
**Fuhrman grade**				
1 + 2	79	33	46	0.047
3 + 4	51	32	19	
**Surgery type**				
Open surgery	34	13	21	0.162
Laparoscopic surgery	96	52	44	

*Note*: Chi‐square analysis between high/low hsa_circ_0057105 patient groups and clinicopathological characteristics in 130 ccRCC patients. Median expression of hsa_circ_0057105 was used as the cut‐off point.

**TABLE 2:** Univariate and multivariate Cox regression analyses of different parameters on overall survival.

Parameter	Univariate analysis		Multivariate analysis	
	HR (95% CI)	*p‐*Value	HR (95% CI)	*p*‐Value
Age	1.050 (1.017‐1.084)	0.003	1.039 (1.004‐1.075)	0.026
Gender (female vs. male)	1.406 (0.558‐3.543)	0.470	–	–
Clinical (TNM) stage (II–III vs. I)	2.082 (1.171‐3.699)	0.012	1.068 (0.570‐2.002)	0.837
Fuhrman (3 + 4 vs. 1 + 2)	2.955 (1.584‐5.514)	<0.001	2.119 (1.089‐4.126)	0.027
Surgery type (Lapar. vs. open)	0.656 (0.307‐1.401)	0.277	–	–
Hsa_circ_0057105 expression (High vs. low)	3.498 (1.636‐7.477)	0.001	3.028 (1.346‐6.812)	0.007

*Note*: Univariate and multivariate Cox regression analyses of different parameters (age, gender, TNM stage, Fuhrman grade, surgery style and hsa_circ_0057105 expression) on overall survival (OS).

Abbreviations: CI, confidence interval; HR, hazard ratio.

**TABLE 3:** Univariate and multivariate Cox regression analyses of different parameters on recurrent‐free survival.

Parameter	Univariate Analysis		Multivariate Analysis	
	HR (95%CI)	*P* Value	HR (95%CI)	*P* Value
Age	1.047 (1.014–1.081)	0.005	1.037 (1.003–1.072)	0.031
Gender (Female vs. male)	1.796 (0.670–4.810)	0.244	–	–
Clinical (TNM) stage (II–III vs. I)	1.854 (1.032–3.329)	0.039	1.100 (0.573–2.113)	0.774
Fuhrman (3 + 4 vs. 1 + 2)	2.587 (1.417–4.721)	0.002	1.923 (1.012–3.652)	0.046
Surgery type (Lapar. vs. open)	1.245 (0.683–2.269)	0.475	–	–
Hsa_circ_0057105 expression (High vs. low)	2.449 (1.273–4.710)	0.007	2.123 (1.038–4.340)	0.039

*Note*: Univariate and multivariate Cox regression analyses of different parameters (age, gender, TNM stage, Fuhrman grade, surgery style and hsa_circ_0057105 expression) on recurrent‐free survival (RFS).

Abbreviations: CI, confidence interval; HR, hazard ratio.

### 3.4 Hsa_circ_0057105 promotes RCC cell aggressiveness in vitro

**FIGURE 4:**
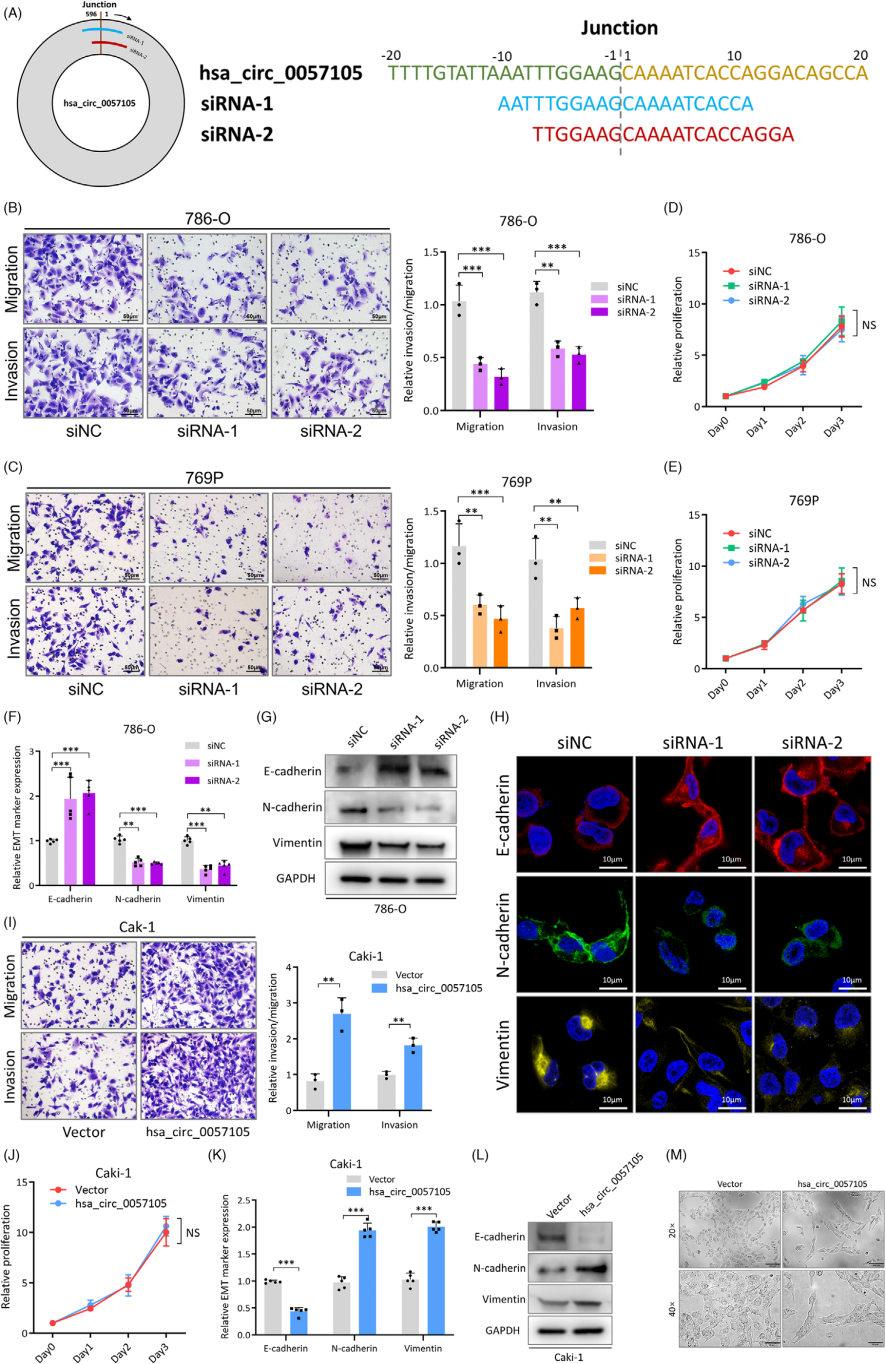
Hsa_circ_0057105 promotes renal cell carcinoma (RCC) cell aggressiveness in vitro. (A) Scheme and sequences of specific siRNAs targeting the back‐splicing junction of hsa_circ_0057105. (B and C) Representative images (left) and quantification (right) data of Transwell migration/invasion assay of 786‐O/769P cells with siNC/siRNA‐1/siRNA‐2 treatment. Cell number was determined by counting five random fields under high power microscope. Data were normalized to the siNC group. (D and E) Proliferative of 786‐O/769P cells with siNC/siRNA‐1/siRNA‐2 treatment. Proliferation was determined by CCK8 assay. Levels were normalized to siNC, day 0. (F) RNA levels of epithelial‐mesenchymal transition (EMT) markers (E‐cad, N‐cad and Vimentin) in 786‐O cells with siNC/siRNA‐1/siRNA‐2 treatment. Expression was determined by qRT‐PCR and normalized to the siNC group. GAPDH was used as an internal control. (G) Protein levels of EMT markers (E‐cad, N‐cad and Vimentin) in 786‐O cells with siNC/siRNA‐1/siRNA‐2 treatment. GAPDH was used as an internal control. (H) IF experiment showing EMT markers (E‐cad, N‐cad and Vimentin) expression in 786‐O cells with siNC/siRNA‐1/siRNA‐2 treatment. (I) Representative images (left) and quantification (right) data of Transwell migration/invasion assay of Caki‐1 cells with vector/hsa_circ_0057105 overexpression treatment. Cell number was determined by counting five random fields under high power microscope. Data were normalized to the vector group. (J) Proliferative of Caki‐1 cells with vector/hsa_circ_0057105 overexpression treatment. Proliferation was determined by CCK8 assay. Levels were normalized to vector, day 0. (K) RNA levels of EMT markers (E‐cad, N‐cad and Vimentin) in Caki‐1 cells with vector/hsa_circ_0057105 overexpression treatment. Expression was determined by qRT‐PCR and normalized to the vector group. GAPDH was used as an internal control. (L) Protein levels of EMT markers (E‐cad, N‐cad and Vimentin) in Caki‐1 cells with vector/hsa_circ_0057105 overexpression treatment. GAPDH was used as an internal control. (M) Representative images of Caki‐1 cell morphology under bright field microscopy with vector/hsa_circ_0057105 overexpression treatment. Data are presented as mean ± SD, *n* = 3.

To further investigate the functional role of hsa_circ_0057105 in RCC cells, two siRNAs specific to the back‐splicing site were constructed (Figure 4A). Transfection experiments confirmed their efficiency at knocking down hsa_circ_0057105 without altering the expression of its linear form (Figure S2A). Consistent with its EMT regulation function, RCC cells transfected with siRNA had a lower migration and invasion ability (Figure 4B,C). However, hsa_circ_0057105 had no influence on RCC cell proliferation (Figure 4D,E and Figure S2B,C). Since hsa_circ_0057105 is associated with EMT activation, EMT markers were assessed in knock‐down RCC cells. As predicted, in hsa_circ_0057105 knock‐down cells, the epithelial marker, E‐cad, was consistently elevated, while the mesenchymal markers, N‐cad and Vimentin, were downregulated (Figure 4F‐H). Hsa_circ_0057105 overexpression (Figure S2D) in RCC cells caused them to become more aggressive (Figure 4I); however cell proliferation remained unaltered (Figure 4J and Figure S2E). EMT markers (E‐cad, N‐cad and Vimentin) had consistent changes after hsa_circ_0057105 overexpression (Figure 4K,L and Figure S2F). Also, hsa_circ_0057105 overexpressed cells showed a more elongated and spinal‐like morphology that was consistent with EMT changes (Figure 4 M)

### 3.5 Hsa_circ_0057105 serves as a sponge for miR‐557 in RCC cells

**FIGURE 5:**
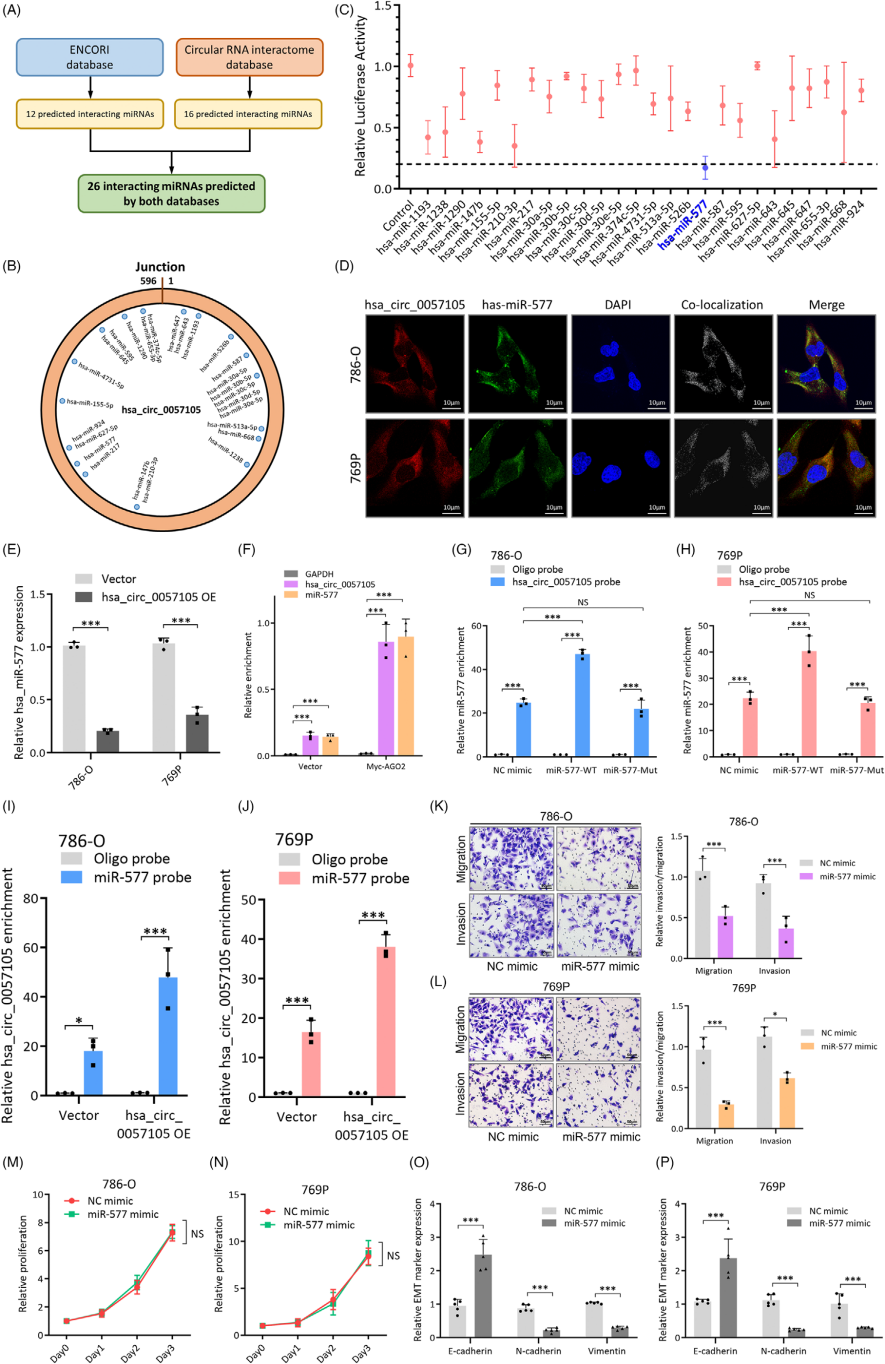
Hsa_circ_0057105 serves as a sponge for miR‐557 in renal cell carcinoma (RCC) cells. (A) Flowchart of circRNA‐miRNA interaction prediction. (B) Scheme of miRNAs with potential hsa_circ_0057105‐binding capacity mapped to the hsa_circ_0057105 sequence. (C) Luciferase reporter assay screening of the 26 predicted miRNAs. Levels were normalized NC mimic control group. Dash line indicated 20% intensity. (D) FISH co‐localization experiment detecting the subcellular localization of hsa_circ_0057105 (Cy3‐labelled) and miR‐577 (FAM‐labelled) in 786‐O and 769P cells. Nuclei were stained with DAPI. Co‐localization was determined by the Colocalization Finder plugin in ImageJ software. (E) RNA levels of miR‐577 in 786‐O/769P cell with vector/hsa_circ_0057105 overexpression treatment. Expression was determined by qRT‐PCR and normalized to the vector group. GAPDH was used as an internal control. (F) AGO2 RIP assay detecting the binding of hsa_circ_0057105 and miR‐577 to AGO2. Myc‐AGO2 indicated AGO2 overexpression group. Expression was determined by qRT‐PCR and normalized to the GAPDH group. (G and H) RNA pull‐down assay with a biotin‐labelled hsa_circ_0057105 probe in 786‐O and 769P cells with NC mimic, miR‐577 wild‐type sequence (WT) and miR‐577 mutant sequence (Mut) treatment. Expression was determined by qRT‐PCR and normalized to the oligo probe group. (I and J) RNA pull‐down assay with a biotin‐labelled miR‐577 probe in 786‐O and 769P cells with vector/hsa_circ_0057105 overexpression treatment. Expression was determined by qRT‐PCR and normalized to the oligo probe group. (K and L) Representative images (left) and quantification (right) data of Transwell migration/invasion assay of 786‐O/769P cells with NC mimic/miR‐577 mimic treatment. Cell number was determined by counting five random fields under high power microscope. Data were normalized to the NC mimic group. (M and N) Proliferative of 786‐O/769P cells with NC mimic/miR‐577 mimic treatment. Proliferation was determined by CCK8 assay. Levels were normalized to NC mimic, day 0. (O and P) RNA levels of epithelial‐mesenchymal transition (EMT) markers (E‐cad, N‐cad and Vimentin) in 786‐O/769P cells with NC mimic/miR‐577 mimic treatment. Expression was determined by qRT‐PCR and normalized to the NC mimic group. GAPDH was used as an internal control. Data are presented as mean ± SD, *n* = 3.

In both normal and cancer cells, a major function of circRNA is its sponge effect on miRNAs, which further influences the function of different pathways. Given that hsa_circ_0057105 primarily localizes to the cytoplasm, it was predicted that this circRNA may function as a miRNA sponge. Two databases were used to determine the interaction between hsa_circ_0057105 and miRNAs (Figure 5A). Twenty‐six potential interactions were predicted and the binding sites were mapped (Figure 5B). A luciferase reporter screening assay was conducted to validate the interactions (Figure S3A). Of the 26 miRNAs, miR‐577 showed the most significant interaction with hsa_circ_0057105 (Figure 5C). FISH analysis also showed co‐localization of miR‐577 and hsa_circ_0057105 (Figure 5D). The expression of miR‐577 was significantly lower in hsa_circ_0057105 overexpressed RCC cells than those receiving the control vector (Figure 5E). In addition, the AGO2 pull‐down experiment confirmed that both hsa_circ_0057105 and miR‐577 could bind to AGO2, which is a premise for circRNA‐miRNA interactions (Figure 5F). To further investigate this interaction, a hsa_circ_0057105‐specific probe was constructed, and an RNA pull‐down assay confirmed its validity (Figure S3B,C). A significant amount of miR‐577 was pulled down with the probe, and the levels increased further following the addition of a miR‐577 mimic but remained unchanged when a miR‐577 mutant mimic was used (Figure 5G,H and Figure S3D). Similarly, when a miR‐577 probe was used in the RNA pull‐down assay, a large amount of hsa_circ_0057105 was pulled down (Figure 5I,J and Figure S3E). Phenotypic studies showed that miR‐577 presented as a tumour‐suppressive miRNA. The migration and invasion ability of RCC cells was significantly lower after transfection with a miR‐577 mimic, but the proliferation rate remained unchanged (Figure 5K–N). Also, miR‐577 exerted potent inhibitive effect on EMT activation of RCC cells (Figure 5O,P and Figure S3F,G). These findings supported a role for miR‐577 as a target of the hsa_circ_0057105 sponge effect.

### 3.6 COL1A1 and VDAC2 are direct targets of miR‐557 in RCC cells

**FIGURE 6:**
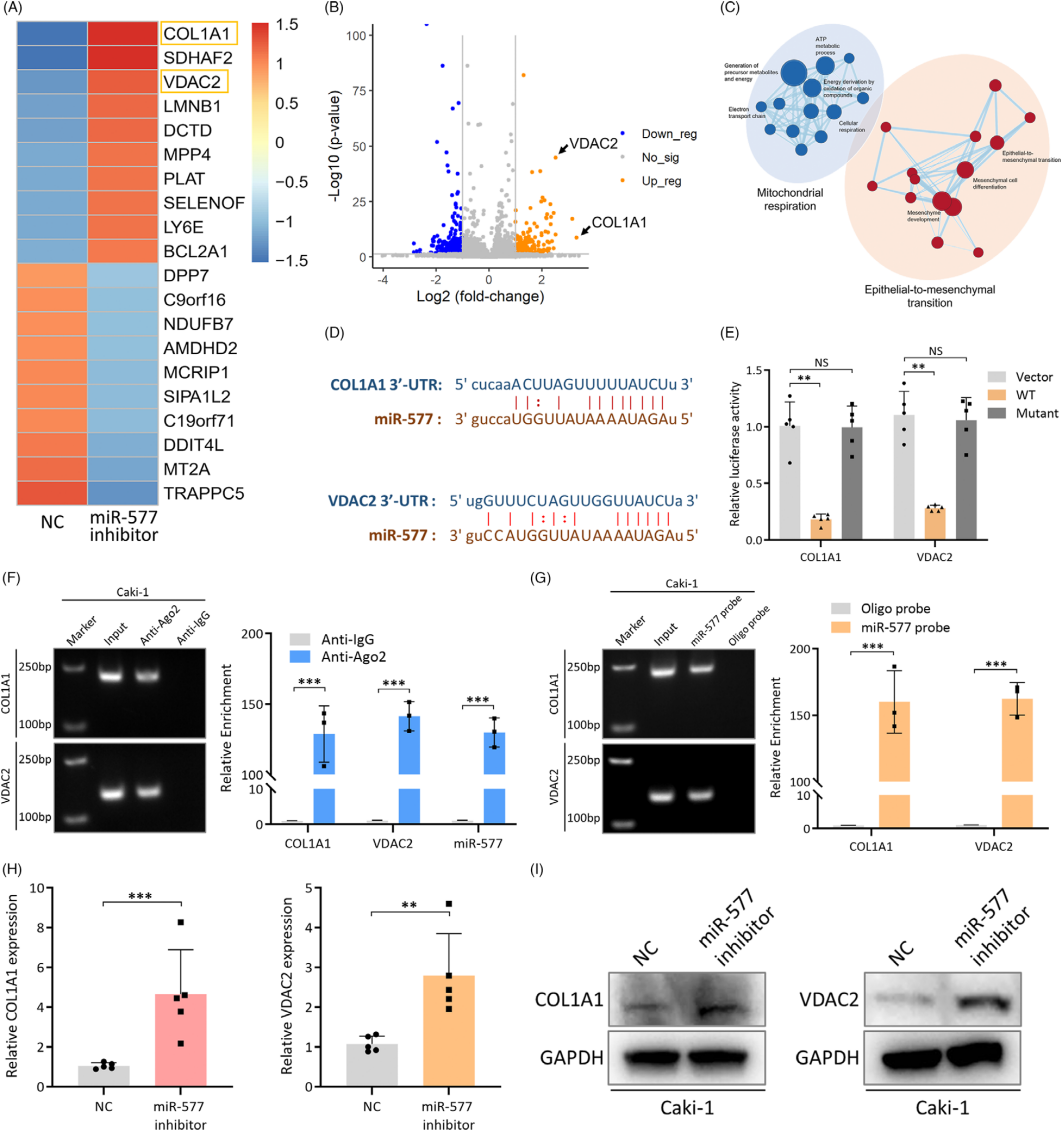
COL1A1 and VDAC2 are direct targets of miR‐557 in renal cell carcinoma (RCC) cells. (A) Heatmap of top 20 up/down‐regulated protein coding genes in Caki‐1 cells with miR‐577 inhibitor treatment compared to NC mimics. (B) Volcano plot of significant protein coding genes in the sequencing dataset (>2‐fold change, *p* < 0.05). Orange dots, blue dots and grey dots indicate up‐regulated, down‐regulated and not significant genes, respectively. (C) Network map showing pathways that were significantly enriched in the miR‐577 inhibitor group. Nodes represent pathways and edges represent shared genes between pathways. (D) Sequences of miR‐577 binding to the 3′‐UTRs of COL1A1 and VDAC2. (E) Luciferase reporter assay of miR‐577 binding to the 3′‐UTRs of COL1A1 and VDAC2. 293T cells were transfected with miR‐577 mimic and different luciferase reporter plasmids. Vector‐control empty plasmid, WT‐palsmids containing wide‐type sequences of COL1A1 or VDAC2, mutant‐palsmids containing mutant sequences of COL1A1 or VDAC2. (F) AGO2 RIP assay detecting the binding of miR‐577 and COL1A1/VDAC2 to AGO2 in Caki‐1 cells. Expression was determined by qRT‐PCR and normalized to the IgG group. (G) RNA pull‐down assay with a biotin‐labelled miR‐577 probe in Caki‐1 cells. Expression was determined by qRT‐PCR and normalized to the oligo probe group. (H) RNA levels of COL1A1 and VDAC2 in Caki‐1 cells with NC mimics/miR‐577 inhibitor treatment. Expression was determined by qRT‐PCR and normalized to the vector group. GAPDH was used as an internal control. (I) Protein levels of COL1A1 and VDAC2 in Caki‐1 cells with NC mimics/miR‐577 inhibitor treatment. GAPDH was used as an internal control. Data are presented as mean ± SD, *n* = 3.

To identify the genes that are regulated by miR‐577, RNA sequencing was performed using miR‐577 inhibitor‐treated and control cells. The top 20 significant differentially expressed genes are presented in a heatmap (Figure 6A) and a volcano plot (Figure 6B). GSEA analysis discovered that miR‐577 and its target genes were involved in EMT and mitochondrial metabolism (Figure 6C). Since the major function of miRNA is to regulate the expression of target genes by binding and degrading their mRNA, we decided to focus on genes that were up‐regulated after miR‐577 inhibitor treatment. Within the top 10 up‐regulated protein‐coding genes, three were significantly associated with patient survival in TCGA KIRC dataset (Figure S4A), including collagen type I alpha 1 chain (COL1A1), voltage dependent anion channel 2 (VDAC2) and deoxycytidylate deaminase (DCTD). Further sequence analysis discovered that only COL1A1 and VDAC2 had specific sequences located in their 3′‐ untranslated region (3′‐UTR), which could be directly regulated by miR‐577 in a posttranscriptional manner (Figure 6D). Moreover, gene ontology (GO) analysis demonstrated that COL1A1 and VDAC2 were related to extracellular matrix organization and cellular metabolism (Figure S4B,C), which reflected the major functions of miR‐577 (EMT and mitochondrial metabolism, Figure 6C). Luciferase reporter assay confirmed the regulatory role of miR‐577 on COL1A1 and VDAC2 (Figure 6E). AGO2 pull‐down assay confirmed that all of the three RNAs could bind to AGO2 protein, which is essential for the regulatory process (Figure 6F). Moreover, RNA pull‐down assay confirmed the direct interactions among the three RNAs (Figure 6G). Finally, miR‐577 was shown to regulate COL1A1 and VDAC2 on both the RNA and protein levels (Figure 6H,I). These findings indicated that COL1A1 and VDAC2 were direct targets of miR‐577.

### 3.7 COL1A1 controls EMT activation in RCC cells

**FIGURE 7:**
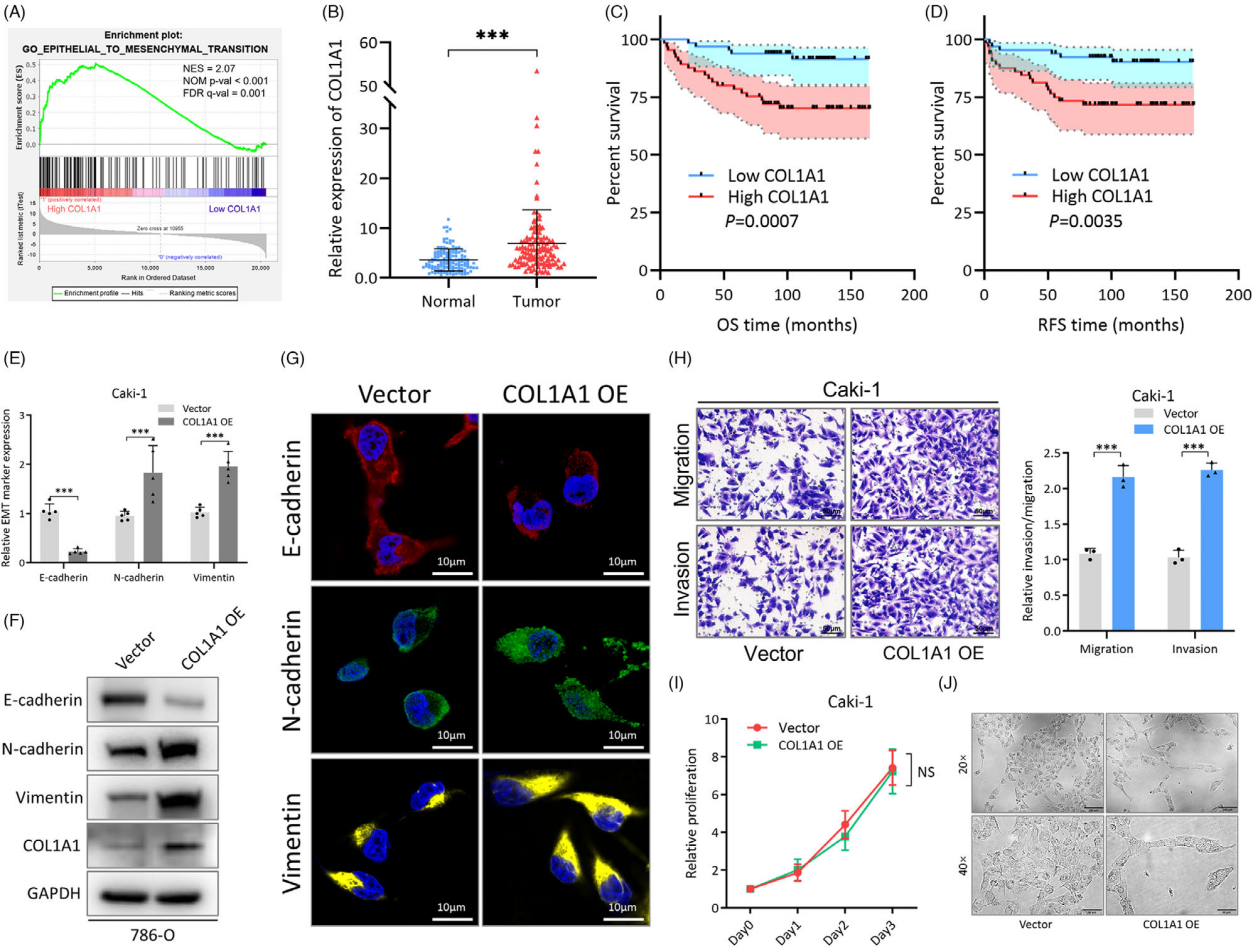
COL1A1 controls epithelial‐mesenchymal transition (EMT) activation in renal cell carcinoma (RCC) cells. (A) GSEA analysis of EMT pathway activation in COL1A1 high/low patient groups of TCGA KIRC dataset. NES‐normalized enrichment score, NOM p‐val‐nominal p value, FDR q‐val‐false discovery rate q value. (B) Expression of COL1A1 in 130 ccRCC patient tumour and normal adjacent tissue samples. (C and D) Survival analysis of OS and RFS in patient groups with high and low COL1A1 expression (median expression as cut‐off), presented as Kaplan–Meier curve. Significance was determined by Log‐rank test. (E) RNA levels of EMT markers (E‐cad, N‐cad and Vimentin) in Caki‐1 cells with vector/COL1A1 overexpression treatment. Expression was determined by qRT‐PCR and normalized to the vector group. GAPDH was used as an internal control. (F) Protein levels of EMT markers (E‐cad, N‐cad and Vimentin) in Caki‐1 cells with vector/COL1A1 overexpression treatment. GAPDH was used as an internal control. (G) IF experiment showing EMT markers (E‐cad, N‐cad and Vimentin) expression in Caki‐1 cells with vector/COL1A1 overexpression treatment. (H) Representative images (left) and quantification (right) data of Transwell migration/invasion assay of Caki‐1 cells with vector/COL1A1 overexpression treatment. Cell number was determined by counting five random fields under high power microscope. Data were normalized to the vector group. (I) Proliferative of Caki‐1 cells with vector/COL1A1 overexpression treatment. Proliferation was determined by CCK8 assay. Levels were normalized to vector, day 0. (J) Representative images of Caki‐1 cell morphology under bright field microscopy with vector/COL1A1 overexpression treatment. Data are presented as mean ± SD, *n* = 3.

COL1A1 is a major component of the extracellular matrix that can regulate the activation of EMT. GSEA analysis using TCGA KIRC dataset confirmed that COL1A1 was involved in EMT (Figure 7A), and higher expression significantly correlated with unfavorable OS and RFS (Figure S5A,B). Similarly, in our patient cohort, higher expression of COL1A1 was detected in tumour samples, and it was associated with poor OS and RFS (Figure 7B–D). Correlation analysis showed that in TCGA KIRC dataset, COL1A1 was tightly related to E‐cad, N‐cad and Vimentin (Figure S5C). Further assessment of these EMT markers in COL1A1 overexpressed RCC cells showed lower E‐cad and higher N‐cad and vimentin expression, which was consistent with EMT activation (Figure 7E–G). COL1A1 overexpression enhanced RCC cell migration and invasion (Figure 7H) but had no effect on the proliferation rate (Figure 7I). RCC cells also became elongated and spinal‐like after COL1A1 overexpression, indicating they were in a mesenchymal status (Figure 7J).

### 3.8 VDAC2 increases sensitivity to the ferroptosis inducer, erastin, in RCC cells

**FIGURE 8:**
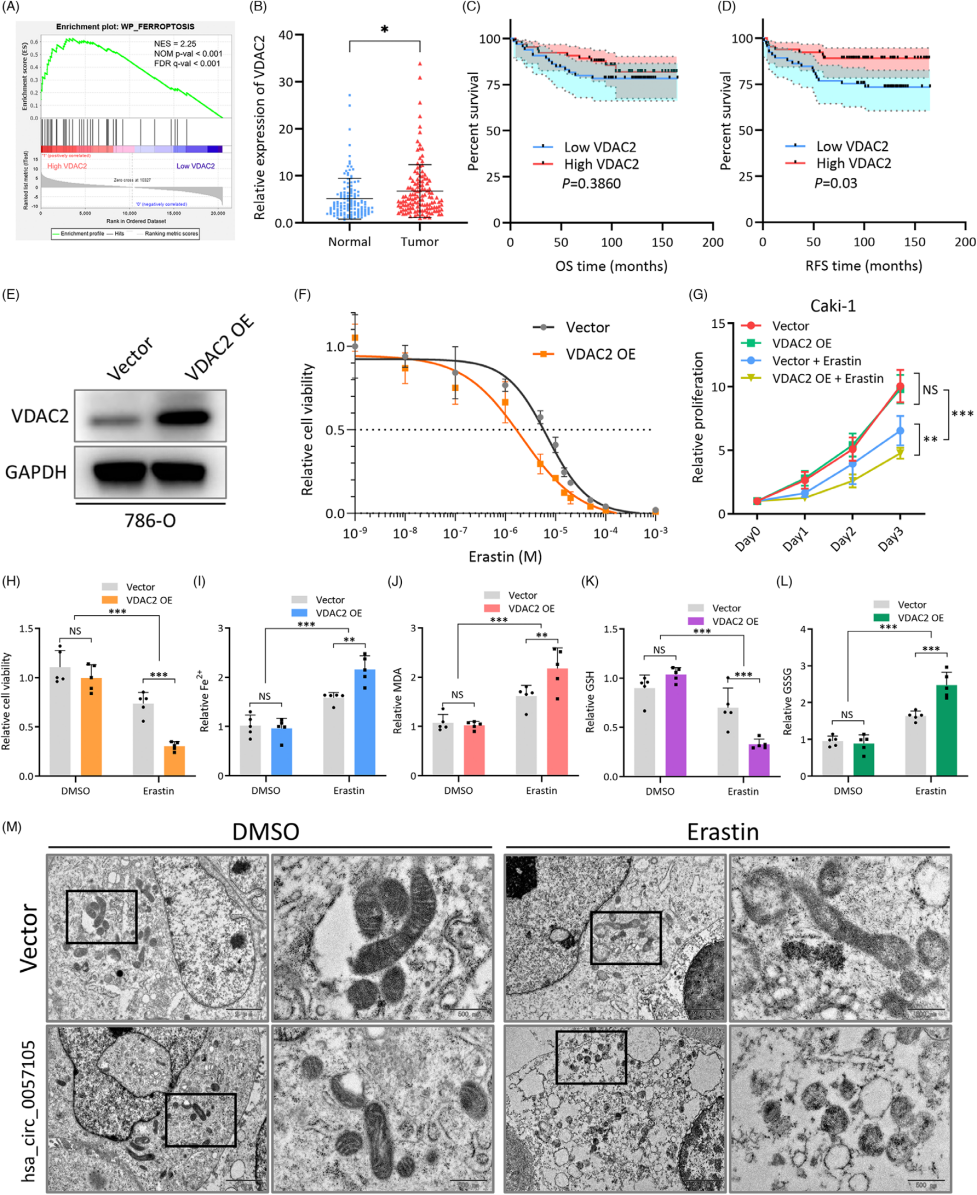
VDAC2 increases sensitivity to the ferroptosis inducer, erastin, in renal cell carcinoma (RCC) cells. (A) GSEA analysis of ferroptosis pathway in VDAC2 high/low patient groups of TCGA KIRC dataset. NES‐normalized enrichment score, NOM p‐val‐nominal *p* value, FDR q‐val‐false discovery rate q value. (B) Expression of VDAC2 in 130 ccRCC patient tumour and normal adjacent tissue samples. (C and D) Survival analysis of OS and RFS in patient groups with high and low VDAC2 expression (median expression as cut‐off), presented as Kaplan–Meier curve. Significance was determined by Log‐rank test. (E) Confirmation of VDAC2 overexpression protein level by western blot. (F) Dose‐response curve of erastin in Caki‐1 cells with vector/VDAC2 overexpression treatment. Dash line indicates IC_50_. (G) Proliferative of Caki‐1 cells with vector, VDAC2 overexpression, vector + erastin and VDAC2 overexpression + erastin treatment. Proliferation was determined by CCK8 assay. Levels were normalized to Vector, day 0 group. (H–L) Detection of cellular viability, Fe^2+^, MDA, GSH and GSSG in Caki‐1 cells with vector/VDAC2 overexpression and DMSO/erastin (5 μM) treatment. Data were normalized to the vector, DMSO group. (M) Representative pictures of subcellular structures from Caki‐1 cells with vector/VDAC2 overexpression and DMSO/erastin (5 μM) treatment. Pictures were captured with transmission electron microscopy. Black rectangles indicate mitochondria. Data are presented as mean ± SD, *n* = 3.

Another miR‐577 targeting gene, VDAC2, is a voltage‐dependent anion channel that controls the exchange of ions and metabolites between the cytosol and the mitochondrion.^23^ Previous studies have reported that VDAC2 can regulate the ferroptosis sensitivity of melanoma cells.^23^, ^24^ GSEA analysis of TCGA KIRC data also found that higher VDAC2 expression is associated with elevated ferroptosis (Figure 8A). Survival analysis indicated that TCGA KIRC patients with high VDAC2 expression had longer OS and RFS (Figure S6A,B). This may be because RCC cells with higher VDAC2 expression are vulnerable to ferroptosis‐induced oxidative damage. However, in our patient samples, although VDAC2 was differentially expressed in tumour samples and was associated with RFS, no significance was observed in OS (Figure 8B–D). VDAC2 overexpression (Figure 8E) did not directly induce ferroptosis and had no impact on proliferation or intercellular iron, malondialdehyde (MDA), glutathione (GSH), and glutathione disulfide (GSSG) levels (Figure 8F–L). However, when erastin, a ferroptosis inducer that targets VDAC2,^25^ was added to the cells, a more potent ferroptosis effect was observed in the VDAC2 overexpression group, as illustrated by iron accumulation, MDA overproduction, GSH depletion, and the generation of GSSG (Figure 8F–L). When another ferroptosis inducer, RSL3, was used, there was no change in ferroptosis sensitivity (Figure S6C–H). This is likely because RSL3 primarily targets glutathione peroxidase 4 (GPX4) to induce ferroptosis, not VDAC2.^25^ Also, COL1A1 overexpression had no influence on ferroptosis sensitivity, meaning that this effect was primarily mediated by VDAC2 (Figure S6I–M). Transmission electron microscope (TEM) examination of subcellular structures also demonstrated higher ferroptosis in the VDAC2 overexpression/erastin group, presented as mitochondria shrinkage and rupture (Figure 8 M). These results mainly supported that VDAC2 acted as a ferroptosis potentiator in RCC, but not a direct inducer.

### 3.9 Hsa_circ_0057105 balances EMT and ferroptosis through the miR‐577/COL1A1/VDAC2 axis

**FIGURE 9:**
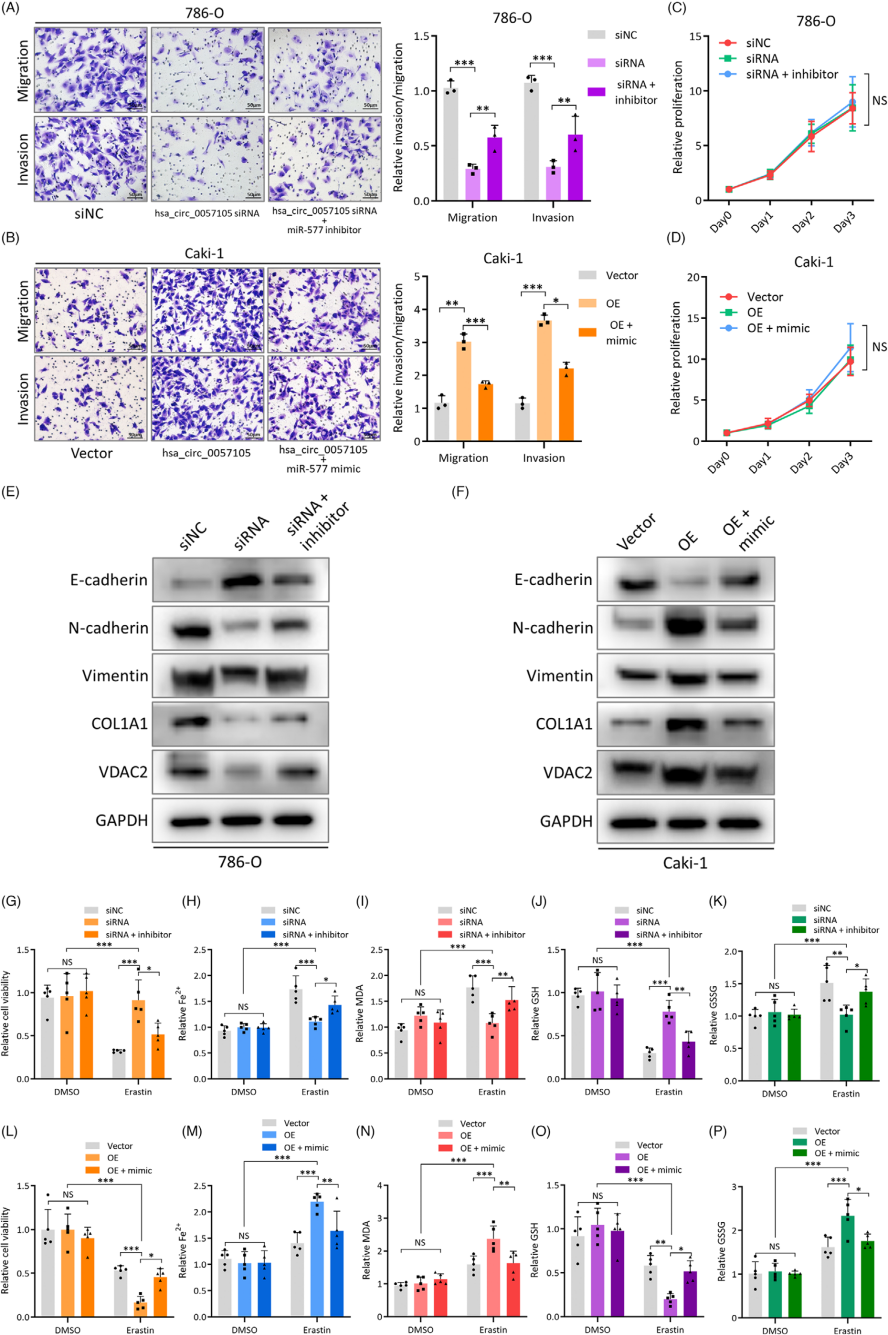
Hsa_circ_0057105 balances epithelial‐mesenchymal transition (EMT) and ferroptosis through the miR‐577/COL1A1/VDAC2 axis. (A and B) Representative images (left) and quantification (right) data of Transwell migration/invasion assay of 786‐O/Caki‐1 cells rescue experiment. Cell number was determined by counting five random fields under high power microscope. Data were normalized to the siNC/vector group. (C and D) Proliferative of 786‐O/Caki‐1 cells rescue experiment. Proliferation was determined by CCK8 assay. Levels were normalized to siNC/vector, day 0. (E and F) Protein levels of EMT markers (E‐cad, N‐cad and Vimentin), COL1A1 and VDAC2 in 786‐O/Caki‐1 cells rescue experiment. GAPDH was used as an internal control. (G–K) Detection of cellular viability, Fe^2+^, MDA, GSH and GSSG in 786‐O cells rescue experiment. Data were normalized to the vector, DMSO group. 10 μM erastin was used. (L–P) Detection of cellular viability, Fe^2+^, MDA, GSH and GSSG in 786‐O cells rescue experiment. Data were normalized to the vector, DMSO group. 5 μM erastin was used. Groupings of rescue experiments: 786‐O cells with siNC/hsa_circ_0057105 siRNA/hsa_circ_0057105 siRNA+miR‐577 inhibitor, and Caki‐1 cells with vector/hsa_circ_0057105 overexpression/hsa_circ_0057105 overexpression+miR‐577 mimic. Data are presented as mean ± SD, *n* = 3.

To investigate the integrity of the regulatory axis, rescue experiments were performed using a miR‐577 inhibitor/mimic in hsa_circ_0057105 knock‐down/overexpressed RCC cell lines. Depletion of hsa_circ_0057105 inhibited 786‐O cell migration and invasion and increased ferroptosis sensitivity and these effects were rescued by adding a miR‐577 inhibitor (Figure 9A,C,G–K). The expression of COL1A1, VDAC2, and EMT markers showed consistent changes, with hsa_circ_0057105 depletion causing EMT deactivation and COL1A1 and VDAV2 down‐regulation that was reversed following the addition of a miR‐577 inhibitor (Figure 9E and Figure S7A). In contrast, when hsa_circ_0057105 overexpression and the miR‐577 mimic were used in Caki‐1 cells, the oppositive results on EMT (Figure 9B,D,F and Figure S7B) and ferroptosis sensitivity (Figure 9L–P) were seen. Adding a miR‐577 inhibitor directly to Caki‐1 cells also caused an increase in ferroptosis sensitivity (Figure S7C–G). Moreover, when RSL3 was applied to these rescue experiments, no alteration of ferroptosis sensitivity was observed, meaning that this effect was specific to erastin and its target VDAC2 (Figure S7H–Q). These findings demonstrated that an intact hsa_circ_0057105 miR‐577/COL1A1/VDAC2 axis is present in RCC and serves to balance EMT and ferroptosis.

### 3.10 Hsa_circ_0057105 balances EMT and ferroptosis of RCC tumours in vivo

**FIGURE 10:**
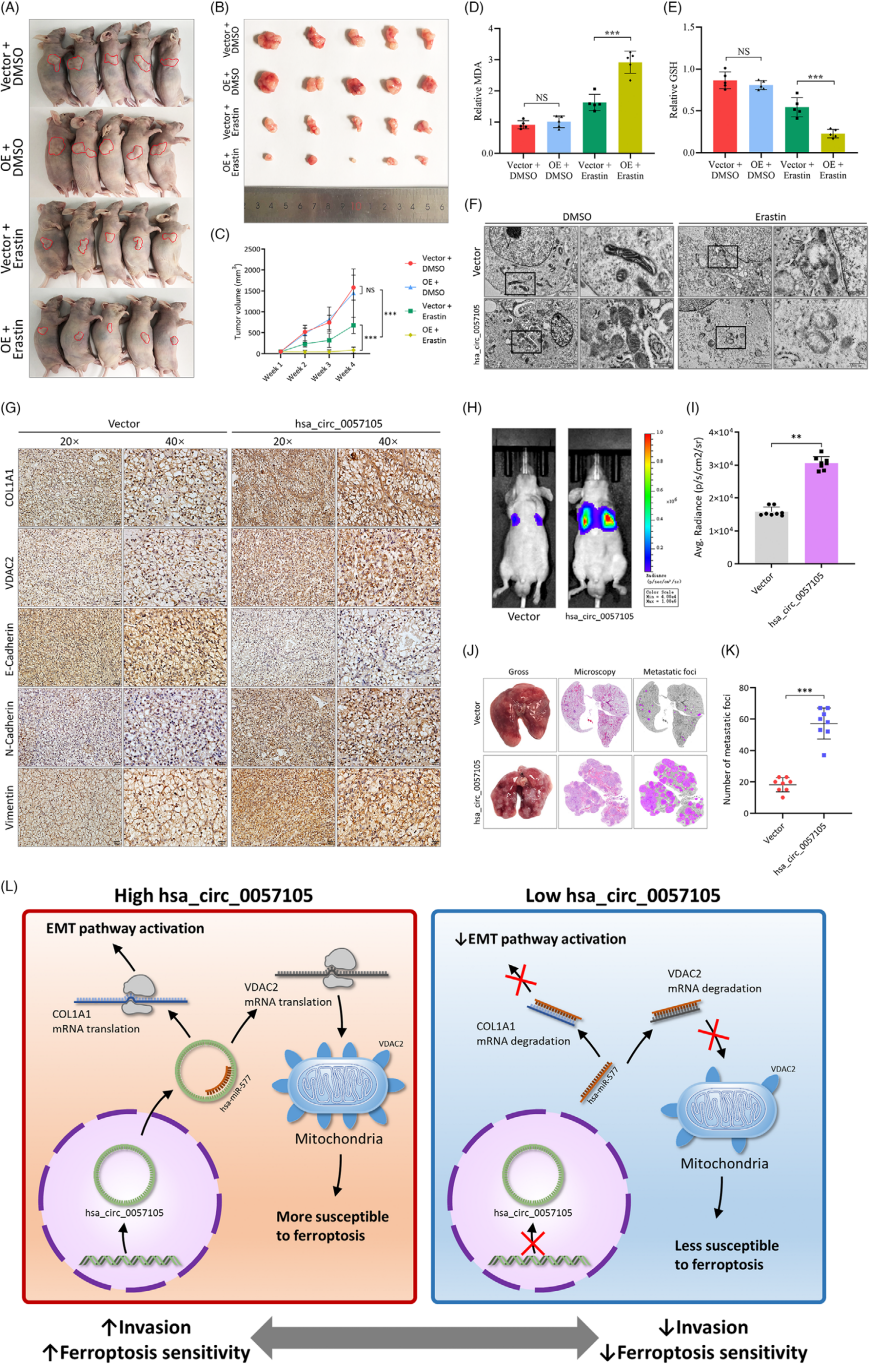
Hsa_circ_0057105 balances epithelial‐mesenchymal transition (EMT) and ferroptosis in renal cell carcinoma (RCC) tumours in vivo. (A) Representative image of nude mice with subcutaneous tumour in different treatment groups: vector + DMSO, hsa_circ_0057105 overexpression + DMSO, vector + erastin, hsa_circ_0057105 overexpression + erastin. Erastin treatment regimen: 15 mg/kg intraperitoneal, twice every other day. (B) Representative images of dissected subcutaneous tumours in different treatment groups. (C) Proliferative curve of subcutaneous tumours in different treatment groups. Data were normalized to the vector + DMSO group. (D and E) Detection of MDA and GSH and GSSG in subcutaneous tumours in different treatment groups. Data were normalized to the vector + DMSO group. (F) Representative pictures of subcellular structures from subcutaneous tumours in different treatment groups. Pictures were captured with transmission electron microscopy. Black rectangles indicate mitochondria. (G) Immunohistochemistry analysis of EMT markers (E‐cad, N‐cad and Vimentin), COL1A1 and VDAC2 of subcutaneous tumours. (H) Representative images of lung metastasis in tail‐vein injection model. Luciferase intensity was shown. (I) Quantification of luciferase intensity in tail‐vein injection model. (J) Representative pictures of lung metastasis with gross view, HE staining microcopy and metastatic foci highlight. (K) Quantification of lung metastatic foci in tail‐vein injection model. (L) Schematic diagram of the function of hsa_circ_0057105 in controlling EMT and ferroptosis.

To evaluate the effect of hsa_circ_0057105 in vivo, Caki‐1 cells transfected with the hsa_circ_0057105 overexpression or control vectors were used in animal models. In the subcutaneous tumour formation model, the overexpression of hsa_circ_0057105 did not alter the growth rate of RCC tumours. However, when erastin was simultaneously injected into the mice, the overexpression group showed more sensitivity, resulting in the slowest tumour growth rate (Figure 10A‐C). Tumours in the overexpression‐erastin combination group also exhibited increased MDA and reduced GSH levels (Figure 10D,E), as well as characteristic ferroptosis‐induced subcellular changes (Figure 10F). Immunohistochemistry (IHC) staining of the subcutaneous tumours showed increased COL1A1, VDAV2, and EMT activation changes in the hsa_circ_0057105 overexpression group (Figure 10G). Although subcutaneous tumours did not cause gross pulmonary metastatic foci in our model (data not shown), higher micro‐metastasis of tumour cells was detected in hsa_circ_0057105 overexpression group, as determined by qRT‐PCR analysis of human GAPDH mRNA in mouse circulation (Figure S8A). In a mouse tail vein injection model, the overexpression of hsa_circ_0057105 caused significantly increased pulmonary colonization of metastatic cells (Figure 10H,I). Microscopic examination of the dissected lungs also revealed more metastatic foci (Figure 10J,K). These findings further strengthened the dual roles of hsa_circ_0057105 on EMT and ferroptosis in vivo.

## 4 DISCUSSION

High‐throughput RNA‐seq and the circRNA‐identifying algorithm have shown that circRNAs are widely expressed in different kinds of human tissues, including solid tumours.^6^ Most studies have focused on the role of circRNAs during cancer and found that they frequently exert oncogenic or tumour‐suppressing functions in tumour cells. In RCC, for example, circSDHC^10^ and circPTCH1^26^ were shown to promote tumour cell aggressiveness, circSNX6^27^ was found to modulate sunitinib resistance, and circ‐AKT3^28^ was shown to inhibit metastasis. While these studies show that circRNAs play an indispensable role in regulating different pathways in RCC, few of them have analyzed the relationship between circRNAs and pathways directly. The current study combined ssGSEA and circRNA‐seq to comprehensively assess the correlation between pathways involved in RCC development and the related circRNAs. EMT differed significantly between tumour and normal adjacent kidney samples, and hsa_circ_0057105 was found to correlate most strongly with EMT activation. EMT is essential for regulating cell plasticity under physiological conditions, but also promotes cancer cell migration and invasion.^29^ Thus, it was proposed that hsa_circ_0057105 play an oncogenic role in RCC. This was confirmed by phenotypic studies and clinical observation. Hsa_circ_0057105 was shown to regulate the expression of COL1A1, a potent EMT regulator in many cancers,^30^, ^31^, ^32^ through its sponge effect on miR‐577.

While no change in RCC cell proliferation was observed when hsa_circ_0057105 was overexpressed or knocked down, this circRNA was shown to regulate the expression of VDAC2, a nuclear‐encoded mitochondrial protein involved in ferroptosis. VDAC2 is a voltage‐dependent anion channel that controls the exchange of ions and metabolites between the cytosol and mitochondria.^23^ Higher expression of VDAC2 is closely linked to ferroptosis sensitivity.^23^, ^24^ Indeed, when VDAC2 was overexpressed, RCC cells demonstrated increased sensitivity to the ferroptosis inducer, erastin. However, when VDAC2‐overexpressed RCC cells were treated with RSL3, no increased sensitivity was observed. This is because erastin targets a specific binding site on VDAC2, while RSL3 primarily inhibits GPX4 in a VDAC‐independent manner.^25^ This indicates that hsa_circ_005710 regulates ferroptosis sensitivity using VDAC2. Interestingly, a previous study found that RSL3 induces VDAC2 carbonylation in fibrosarcoma cells and counters its effect on ferroptosis.^33^ This change was not observed in RCC cells, however, indicating that the carbonylation effect may be tissue specific.

The dual regulatory effect of hsa_circ_0057105 balances EMT and ferroptosis sensitivity in RCC. A previous study has reported that E‐cad regulates ferroptosis in RCC using the Hippo pathway.^34^ This suggests that EMT and ferroptosis may be closely related, and as cancer cells develop a more aggressive EMT phenotype, their resistance to ferroptosis may be lost. This phenomenon was reported in many different types of cancers. In head and neck cancer, manipulation of several EMT markers/drivers, including E‐cad, ZEB1, SIRT1, and the miR‐200 family, causes dramatic changes in ferroptosis sensitivity.^19^ Adrenal adenocarcinoma treatment using histone deacetylase inhibitors causes cells to adopt a mesenchymal phenotype while altering iron metabolism and increasing ferroptosis sensitivity.^21^ This indicates that some cancer cells may have a trade‐off mechanism between proliferation and metastasis.

The special function of hsa_circ_0057105 in RCC makes it a useful target to aid disease surveillance and guide therapy. Patients with a high expression of hsa_circ_0057105 require more frequent follow‐ups for early recurrence detection. If metastasis or unresectable disease occurs, these patients are potential candidates for ferroptosis inducer therapy. However, there are still concerns regarding the use of ferroptosis inducers given that they can effectively inhibit cancer spread while also initiating or worsening other diseases.^35^ Thus, more research is needed to determine the optimal dosage and route of administration of ferroptosis inducers in RCC.

## 5 CONCLUSIONS

This study combines high‐throughput RNA‐seq and the ssGSEA algorithm and illustrates that EMT is highly activated in RCC and is regulated by the oncogenic circRNA, hsa_circ_0057105. Hsa_circ_0057105 can also regulate the expression of COL1A1 and VDAC2 through its sponge effect on miR‐577. COL1A1 is involved in EMT activation while VDAC2 regulates ferroptosis sensitivity. These findings demonstrate that hsa_circ_0057105 modulates the balance of EMT and ferroptosis in RCC and can serve as a potential biomarker for disease surveillance and a target for treatment.

## CONFLICT OF INTEREST STATEMENT

The authors declare no conflict of interest.

## Supporting information

Supporting Information

Supporting Information

Supporting Information

Supporting Information

Supporting Information

## Data Availability

For all data requests, please contact the corresponding author.
