# Supplementary material for: Hsa_circ_0057105 modulates a balance of epithelial‐mesenchymal transition and ferroptosis vulnerability in renal cell carcinoma
Source: Clin Transl Med. 2023 Jul 26;13(8):e1339. doi: 10.1002/ctm2.1339 (PMC10372385; doi:10.1002/ctm2.1339)
Supplement: Supplementary file 1 — Supporting Information [file CTM2-13-e1339-s004.docx]

**Supplemental materials and methods**

**Plasmid and small interfering RNA (siRNA) transfection**

CircRNA overexpression plasmid was synthesized using the pLO-ciR vector (Geneseed Biotech, Guangzhou, China). COL1A1 and VDAC2 overexpression plasmids were synthesized using the pCDH vector (Tsingke, Beijing, China). SiRNAs were designed and synthesized by RiboBio (Guangdong, China). JetPEI reagent (Polyplus-transfection, Illkirch, France) was used for the siRNA transfection and jetPRIME reagent (Polyplus-transfection) was used for the plasmid transfection. siRNAs sequences used in transfection are listed in Table S3.

**Quantitative real-time PCR (qRT-PCR)**

For qRT-PCR, total RNA was first extracted and purified from cells using TRIzol (Invitrogen) according to the manufacturer’s protocol. After measuring the RNA concentration with NanoDrop (ThermoFisher), 1 μg of total RNA was subjected to cDNA reverse transcription using the iScript cDNA Synthesis Kit (Bio-Rad, CA, USA). For miRNA, the linear poly(A) tailed method was utilized to synthesize the first strand of cDNA. Using 2X SYBR Green Pro Taq HS Premix II (AGbio, Hunan, China), the cDNAs were amplified and quantified using a QuantStudio 5 real-time PCR instrument (ThermoFisher). Glyceraldehyde 3-phosphate dehydrogenase (GAPDH) and U6 small nuclear RNA were used as internal normalization controls for mRNA and miRNA respectively. The 2-ΔΔCt method was used to calculate the relative expression of different genes. Primers used in qRT-PCR are listed in Table S3.

**RNase treatment**

RNase treatment was used to test the resistance of different RNAs. Total RNA was extracted from cells as described above and 2 μg aliquots were incubated with or without 3 U/μg RNase R (Lucigen, WI, USA) for 30 min at 37 °C. The digestion products were purified using a RNeasy MinElute cleaning Kit (Qiagen, MD, USA) and subjected to qRT-PCR analysis. The qRT-PCR products were further subjected to electrophoresis on 2% agarose gels and visualized using Safe Green (Biosharp, Anhui, China). Divergent and convergent primers used in this experiment are listed in Table S3.

**Actinomycin D treatment**

Actinomycin D treatment was performed to test the stability of different RNAs. After cells were attached, 2 μg/ml actinomycin D or control dimethyl sulfoxide (DMSO) was added to the culture plates. After certain time points (0, 4, 8, 12, 16, 20, and 24h), cells were harvested and subjected to qRT-PCR.

**Measurement of cDNA and genomic DNA (gDNA)**

Extraction of RNA and reverse transcription of cDNA were performed as described above. gDNA extraction was performed using a kit according to the manufacturer’s protocol (Tiangen, Beijing, China). Both the cDNA and gDNA were subjected to PCR amplification. The PCR products were further subjected to electrophoresis on 2% agarose gels and visualized using Safe Green (Biosharp). Divergent and convergent primers used in this experiment are listed in Table S3.

**Transwell assay**

To perform the transwell assay, cells were first starved with serum-free medium overnight. Cells were then trypsinized and adjusted to a final concentration of 1×10^5^ cells/100μl in serum-free medium. For the transwell migration assay, 100 μl of cells were directly added to the transwell inserts (Corning, NY, USA). For the invasion assay, the inserts were coated with % Matrigel (Corning) and incubated overnight before adding the cells. The inserts were then placed on a 24-well plate (Corning) containing medium supplemented with 10% FBS. After 8h (for migration) and 24h (for invasion), the inserts were collected and fixed with 4% polyformaldehyde (Biosharp). After fixation, 0.4% crystal violet (Biosharp) was used to stain the cells on the inserts. A cotton swab was applied to clean off any cells on the upper side of the inserts that did not migrate or invade. The cells on the lower surface were visualized using an IX83 inverted microscope (Olympus, Tokyo, Japan), and five random high-power fields were captured.

**CCK-8 assay**

To perform the CCK-8 assay, 2×10^3^ cells in 100 μl medium were seeded in each well of a 96-well plate. After the cells were attached, different treatments were applied. At specified time periods, 10 μl of CCK-8 reagent (Biosharp) was added to each well and incubated for 1h. The absorbance of CCK-8 (450nm) was measured using a Varioskan LUX machine (ThermoFisher).

**Western blot**

Cells receiving different treatment conditions were trypsinized and collected. Radioimmunoprecipitation assay (RIPA) buffer (ThermoFisher) supplemented with proteinase inhibitor (Beyotime) was added to lyse the cells and the samples were centrifuged. The protein samples were then subjected to electrophoresis on a sodium dodecyl sulfate-polyacrylamide (SDS-PAGE) gel. The separated proteins were transferred onto a 0.2μm polyvinylidene fluoride membrane (MilliporeSigma, CA, USA). The membrane was blocked with 5% non-fat milk (Cell Signaling Technology, MA, USA) and incubated with specific primary antibodies overnight at 4°C. The membrane was washed thoroughly with PBS, and HRP-conjugated secondary antibody specific for mouse/rabbit IgG was added for 1h at room temperature. Hybridization was detected using a SuperSignal West Pico PLUS (ThermoFisher) on a FluorChem E System (General Electric, MA, USA). Antibodies used in immunoblotting included the following: E-cadherin (20874-1-AP, Proteintech, Wuhan, China), N-cadherin (22018-1-AP, Proteintech), Vimentin (10366-1-AP, Proteintech), COL1A1 (67288-1-Ig, Proteintech), VDAC2 (11663-1-AP, Proteintech), GAPDH (D16H11, Cell Signaling Technology), HRP-conjugated goat anti-rabbit IgG (SA00001-2, Proteintech), HRP-conjugated goat anti-mouse IgG (SA00001-1, Proteintech).

**Luciferase reporter assay**

Wild type or mutant sequences of hsa_circ_0057105/3’-UTR COL1A1/3’-UTR VDAC2 were inserted into the psiCHECK-2 vector. Cells (3×10^3^ 293T) were seeded in each well of 96-well plates and 50 ng of psiCHECK-2 and 5 ng of Renilla vectors (pRL-TK) were used to transfect each well, along with different miRNA mimics. After 48h of transfection, luciferase activity was determined using a dual luciferase reporter assay kit (Promega, WI, USA) on a Varioskan LUX machine (Thermo, MA, USA). In each well, the luciferase activity was normalized to the Renilla control. Critical sequences used in luciferase reporter assay are listed in Table S3.

**Fluorescence in situ hybridization (FISH)**

Cells were seeded onto a glass plate. After attachment, the cells were fixed with 4% polyformaldehyde (Biosharp). Cy3-labeled hsa_circ_0057105, FAM-labeled miR-577, Cy3-labeled 18s, and Cy3-labeled U6 probes were synthesized (RiboBio) and hybridized overnight at 37 °C using a FISH hybridization kit (RiboBio), according to the manufacturer’s protocol. The nuclei were stained with 4',6-diamidino-2-phenylindole (DAPI) for 5 min. Fluorescence images were captured using a FV3000 confocal microscope (Olympus).

**Immunofluorescence (IF) assay**

Cells were seeded onto 15-mm glass-bottom plates (Nest, Jiangsu. China). After the cells attached, they were fixed with 4% polyformaldehyde (Biosharp) for 20 min. After the fixation, cells were further permeabilized with 0.5% Triton X-100/PBS for 15 min and incubated with primary antibodies overnight at 4°C. After another round of washing with PBS, the cells were incubated with fluorescent secondary antibodies conjugated at room temperature for 1 h. The nuclei were stained with DAPI for 5 min. Fluorescence images were captured on an IX83 inverted microscope (Olympus). Antibodies used in IF are as followed: E-cadherin (ab40772, Abcam, Cambridge, UK), N-cadherin (22018-1-AP, Proteintech), Vimentin (D21H3, Cell Signaling Technology), CoraLite488-conjugated goat anti-rabbit IgG (SA00013-2, Proteintech).

**RNA pull-down**

For RNA immunoprecipitation (RIP), Protein A/G Magnetic Beads (Bimake, TX, USA) were used. In brief, the specific antibody was first conjugated to the magnetic beads according to the manufacturer’s protocol. Cells needed for the experiment were then harvested, washed, and sonicated. The antibody-bound magnetic beads were mixed and incubated with the cell lysate overnight at 4°C. The mixture was washed, and the RNA was extracted with TRIzol (Invitrogen) and assessed using qRT-PCR quantification.

For RNA pull-down with a biotinylated probe, streptavidin magnetic beads (Invitrogen) were used. In brief, the biotinylated probes specific for hsa_circ_0057105 or miR-577 were synthesized (RiboBio). The probes were then incubated with streptavidin magnetic beads on a rotator at room temperature for 2h. Cells needed for the experiments were harvested and washed. The probe-bound magnetic beads were mixed and incubated with the cell lysate overnight at 4°C. The mixture was washed, and the RNA was extracted with TRIzol (Invitrogen) and assessed using qRT-PCR quantification.

**Malondialdehyde (MDA) assay**

The level of MDA represents the status of lipid peroxidation in cells. To perform this assay, cell lysis buffer (Beyotime) and an MDA measurement kit (Beyotime) were used, according to the manufacturer’s protocol. In brief, the cells or tissue (10mg) with different treatment conditions were collected and lysed with the buffer. After centrifugation, 100 μl of supernatant was collected and mixed with 200 μl of MDA working solution. The mixture was heated to 100 °C for 15 min, 200 μl of the mixture was transferred to a 96-well plate, and the absorbance at 532 nm was measured using a Varioskan LUX machine (Thermo).

**Iron assay**

Ferrous iron was detected using an iron assay kit (Abcam). In brief, the cells under different treatment conditions were collected and washed with PBS. Next, a 10x volume of iron assay buffer was added to the cells. The cells were then centrifuged and 100 μl of supernatant was collected and mixed with 5 μl of the iron buffer. After a 30 min incubation at 37 °C, 100 μl of the iron probe was added to the reaction and the mixture was incubated for 1h at 37°C in a darkroom. Finally, the absorbance of the mixture was measured at 593 nm using a Varioskan LUX machine (Thermo).

**Glutathione/GSSG assay**

Intracellular glutathione (GSH) and glutathione disulfide (GSSG) was measured using a GSH/GSSG-Glo assay kit (Promega) according to the manufacturer’s protocol. Cells receiving different treatment conditions were first harvested and washed with ice-cold PBS. For the detection of GSH and GSSG, total GSH lysis and oxidized GSH lysis reagents were prepared, respectively, and used to lyse the cells. After briefly shaking, 50 μl of luciferin generation reagent was added to each sample and the mixture was incubated at room temperature for 30 min. Finally, 100 μl of luciferin detection reagent was added to each sample, and luminescence was measured using a Varioskan LUX machine (Thermo).

**Transmission electron microscopy (TEM)**

The cells or tissues were first fixed with a 0.1M phosphate buffer containing 2.5% glutaraldehyde. After washing with phosphate buffer, the samples were post-fixed with 1% buffered osmium for 2h. After dehydration with gradient alcohol and acetone, samples were embedded overnight. Ultrathin sections were cut using a Leica EM UC7 Ultracut microtome (Leica, IL, USA). After uranyl acetate/lead citrate staining and washing, images of the samples were captured using a JEM-1200EX Electron Microscope (JEOL, Tokyo, Japan).
