# Supplementary material for: Hsa_circ_0057105 modulates a balance of epithelial‐mesenchymal transition and ferroptosis vulnerability in renal cell carcinoma
Source: Clin Transl Med. 2023 Jul 26;13(8):e1339. doi: 10.1002/ctm2.1339 (PMC10372385; doi:10.1002/ctm2.1339)
Supplement: Supplementary file 2 — Supporting Information [file CTM2-13-e1339-s005.docx]

**Supplementary information**

**
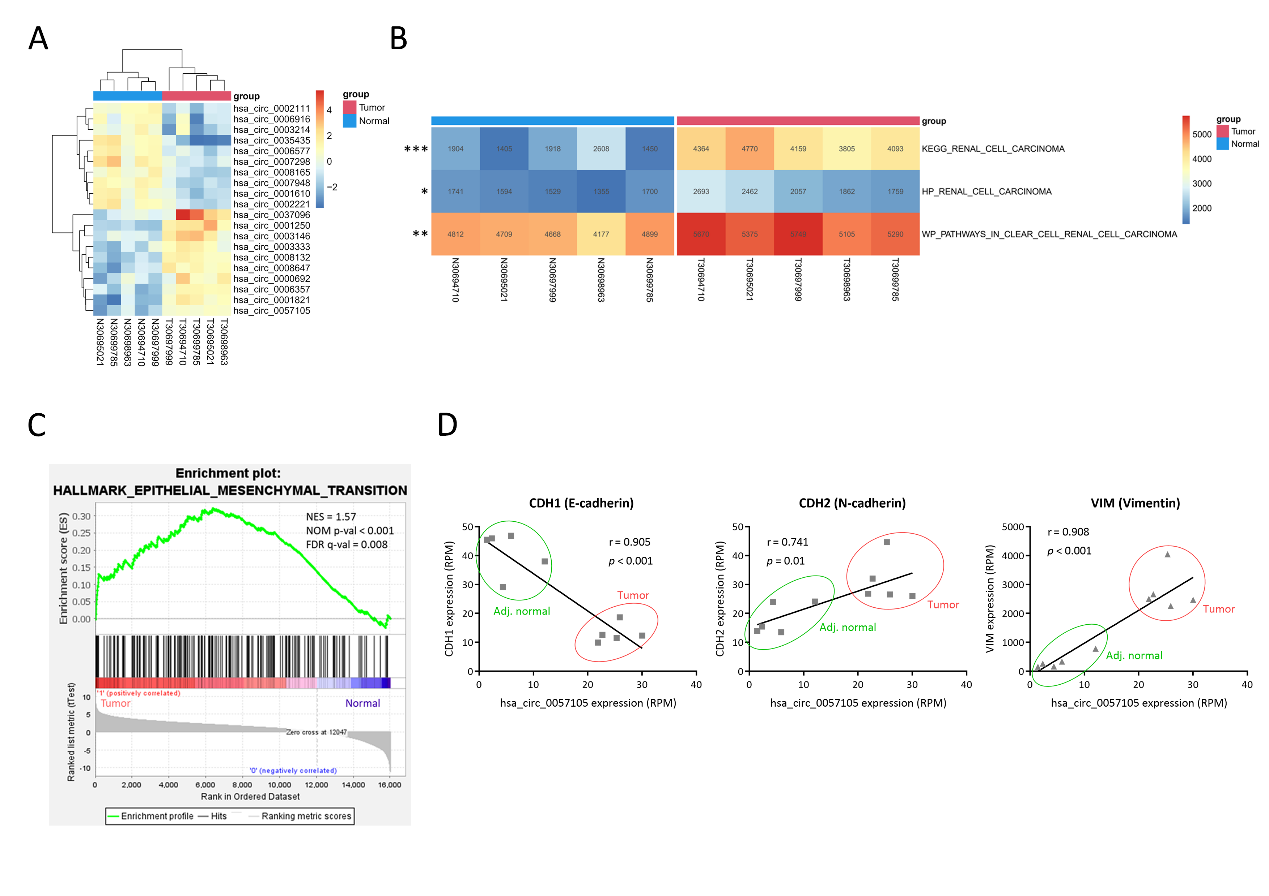
**

**Figure S1. Identification of EMT-related circRNAs using high-throughput sequencing (supplementary information).** **A.** Heatmap of top 20 up/down-regulated circRNAs in 5 pairs of RCC samples sequencing dataset from our institute. **B.** Heatmap of 3 of three RCC tumorigenesis-related pathways in 5 pairs of RCC samples. Asterisks on the left indicate the significance of difference between tumor and normal adjacent samples. Student’s t-test is applied. **C.** GSEA analysis of EMT pathway in tumors compared to normal adjacent samples. NES-normalized enrichment score, NOM p-val-nominal p value, FDR q-val-false discovery rate q value. **D.** Correlation analysis of hsa_circ_0057105 expression and EMT markers (E-cad, N-cad and Vimentin). Red circles and green circles indicate tumor and normal adjacent sample (Adj. normal) datapoints, respectively.

**
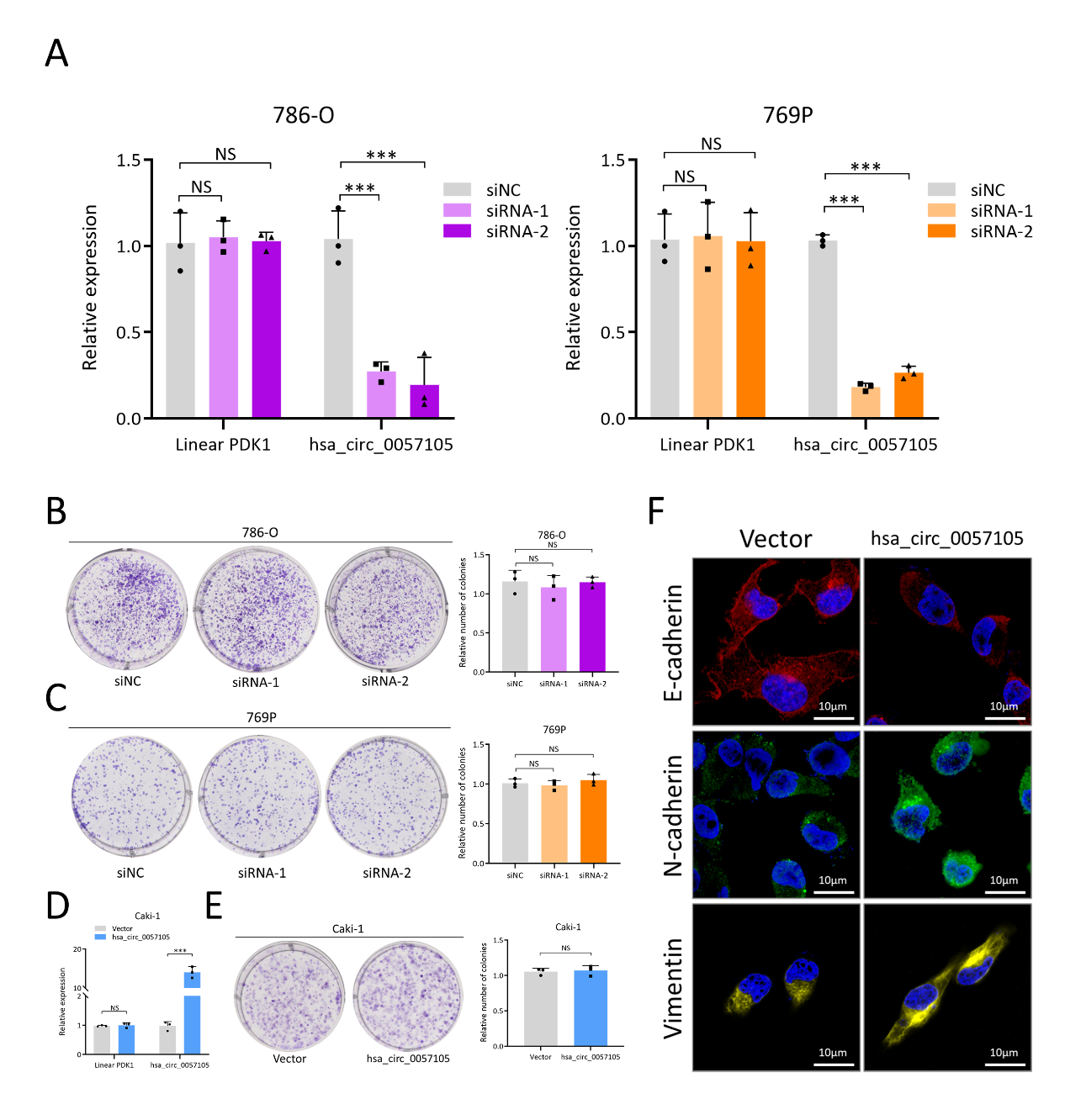
**

**Figure S2. Hsa_circ_0057105 promotes RCC cell aggressiveness *in vitro* (supplementary information).** **A.** qRT-PCR confirmation of knockdown efficiency of hsa_circ_0057105-specific siRNAs in 786-O (left) and 769P (right) cells. Expression was normalized to the siNC group. GAPDH was used as an internal control. **B, C.** Representative pictures (left) and quantification (right) of colony-forming assay of 786-O and 769P cells with siNC/siRNA-1/siRNA-2 treatment. Data was normalized to the siNC group. **D.** qRT-PCR confirmation of overexpression efficiency of hsa_circ_0057105 vector in Caki-1 cells. Expression was normalized to the vector group. GAPDH was used as an internal control. **E.** Representative pictures (left) and quantification (right) of colony-forming assay of Caki-1 cells with vector/ hsa_circ_0057105 overexpression treatment. Data was normalized to the vector group. **F.** IF experiment showing EMT markers (E-cad, N-cad and Vimentin) expression in Caki-1 cells with vector/ hsa_circ_0057105 overexpression treatment. Data are presented as mean ± SD, n = 3.


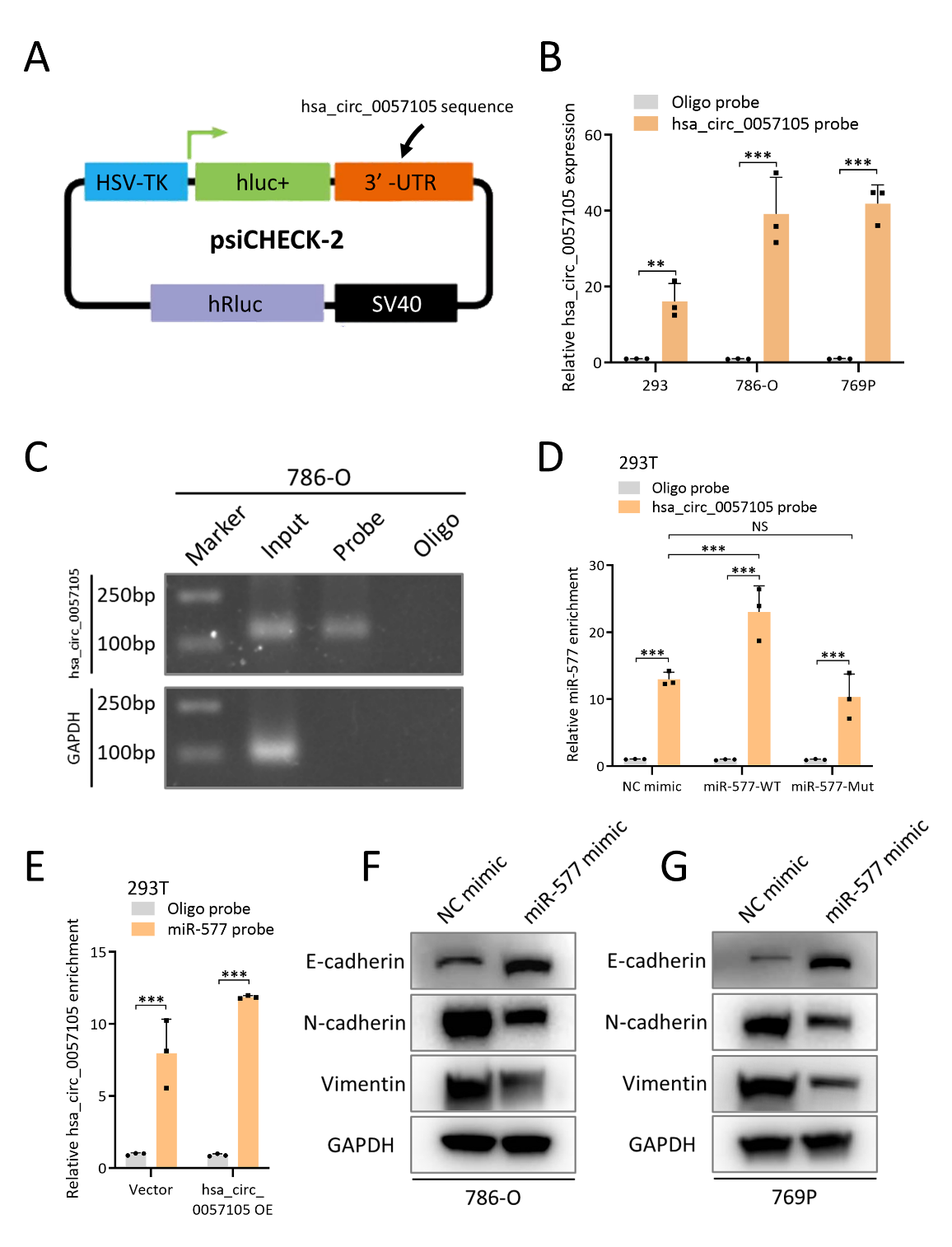


**Figure S3. Hsa_circ_0057105 serves as a sponge for miR-557 in RCC cells (supplementary information).** **A.** Structure of psiCHECK-2 plasmid used in the luciferase reporter assay. HSV-TK, SV40: promoters. hluc+: synthetic firefly luciferase gene. hRluc: synthetic *Renilla* luciferase gene. 3’-UTR: 3’- untranslated region. **B.** RNA pull-down assay confirmation of validity of the biotin-labeled hsa_circ_0057105 probe in 293, 786-O and 769P cells. Expression was determined by qRT-PCR and normalized to the oligo probe group. **C.** DNA electrophoresis of PCR products amplified from RNA pull-down assay of 786-O cells. Probe: biotin-labeled hsa_circ_0057105 probe. Oligo: oligo probe. **D.** RNA pull-down assay with the biotin-labeled hsa_circ_0057105 probe in 293T cells with NC mimic, miR-577 wild-type sequence (WT) and miR-577 mutant sequence (Mut) treatment. Expression was determined by qRT-PCR and normalized to the oligo probe group. **E.** RNA pull-down assay with a biotin-labeled miR-577 probe in 293T cells with vector/hsa_circ_0057105 overexpression treatment. Expression was determined by qRT-PCR and normalized to the oligo probe group. **F, G.** Protein levels of EMT markers (E-cad, N-cad and Vimentin) in 786-O and 769P cells with NC mimic/miR-577 mimic treatment. GAPDH was used as an internal control. Data are presented as mean ± SD, n = 3.

**
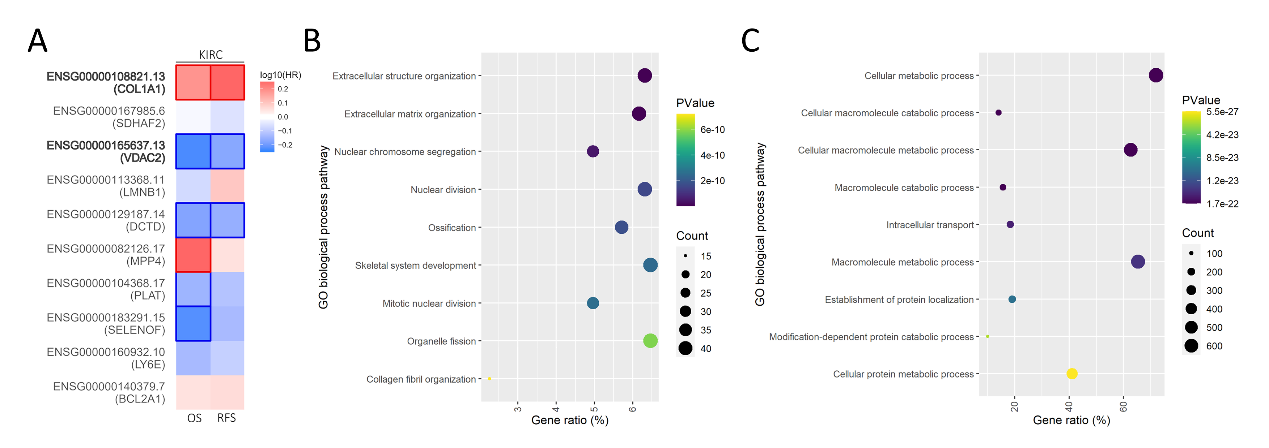
**

**Figure S4. COL1A1 and VDAC2 are direct targets of miR-557 in RCC cells (supplementary information).** **A.** Survival profiling of the top 10 up-regulated protein coding genes in Caki-1 cells with miR-577 inhibitor treatment compared to NC mimics. TCGA KIRC data was used to generate the survival analysis. Red/blue fills indicate correlation with poor/good survival. Solid outlines indicate significance (*p*<0.05). **B, C.** Bubble plots of gene ontology (GO) biological pathway analysis of COL1A1 **(B)** and VDAC2 **(C)**. TCGA KIRC data was used. The most related genes of COL1A1 or VDAC2 were used in the analysis.


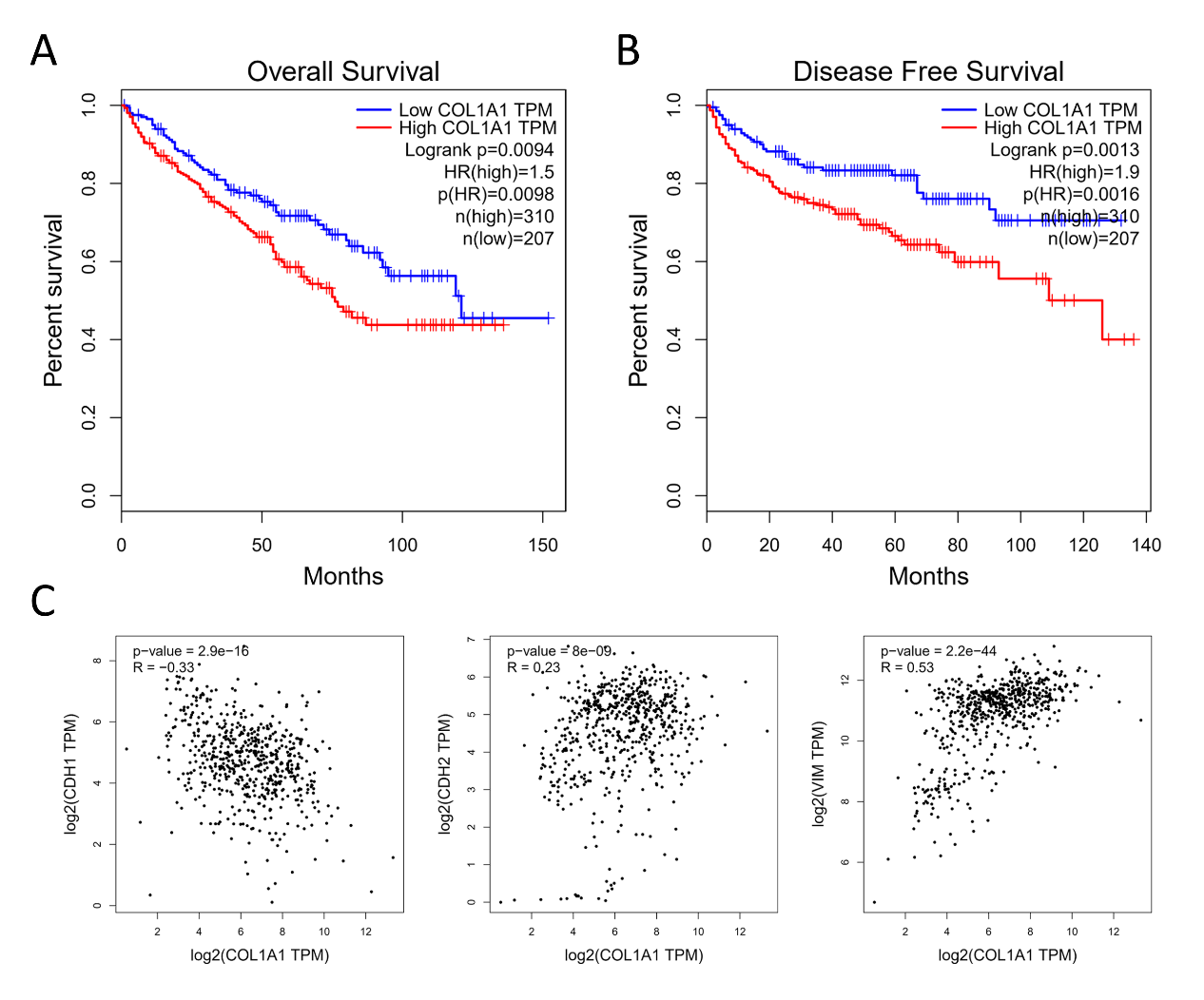


**Figure S5. COL1A1 controls EMT activation in RCC cells (supplementary information).** **A, B.** Survival analysis of OS and RFS in patient groups with high and low COL1A1 expression, presented as Kaplan–Meier curve. TCGA KIRC data was used. **C.** Correlation analysis of COL1A1 and EMT markers (E-cad, N-cad and Vimentin) in TCGA KIRC dataset. R: Pearson correlation coefficient.


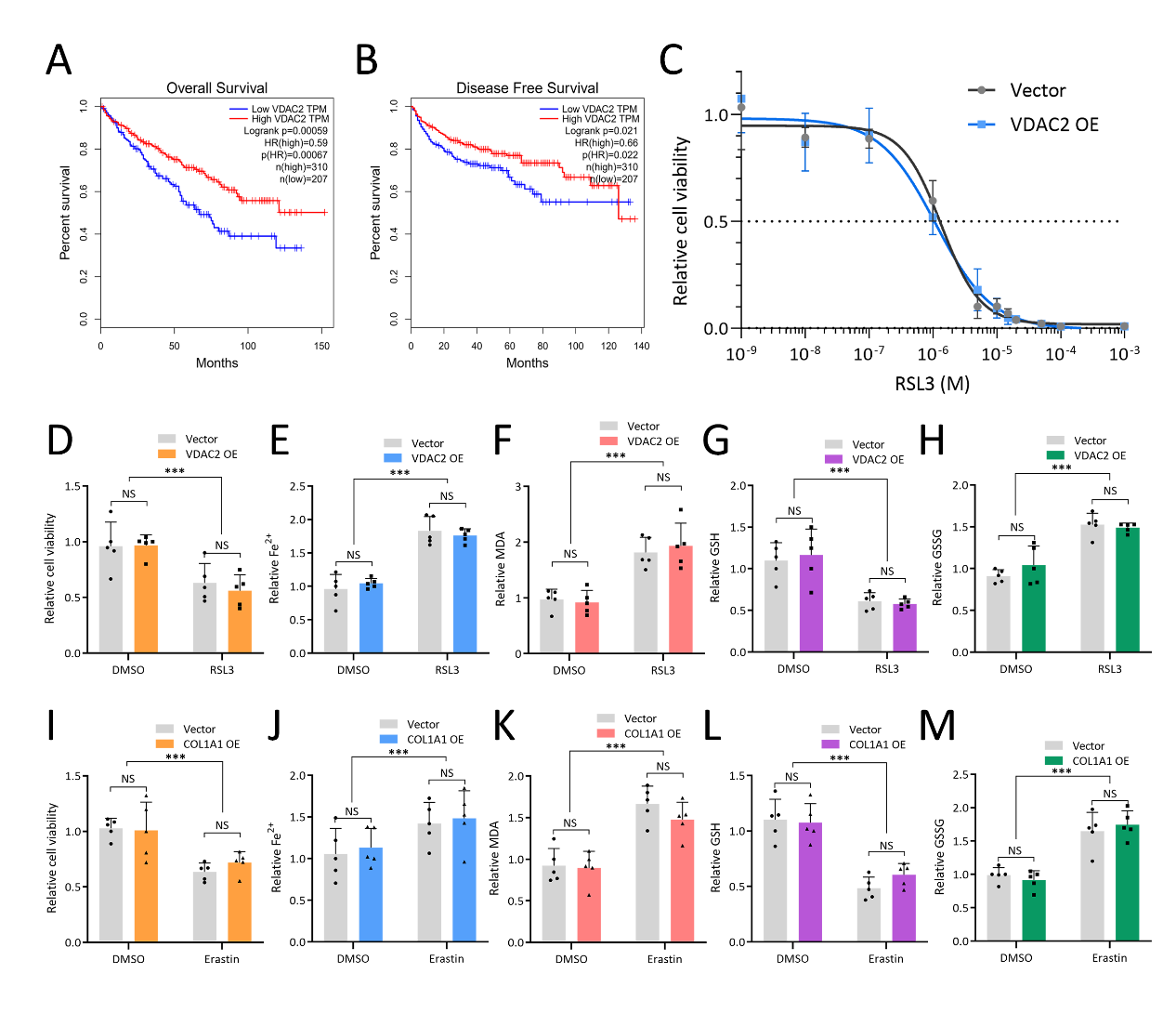


**Figure S6. VDAC2 increases sensitivity to the ferroptosis inducer, erastin, in RCC cells (supplementary information).** **A, B.** Survival analysis of OS and RFS in patient groups with high and low VDAC2 expression, presented as Kaplan–Meier curve. TCGA KIRC data was used. **C.** Dose-response curve of RSL3 in Caki-1 cells with vector/VDAC2 overexpression treatment. Dash line indicates IC_50_. **D-H.** Detection of cellular viability, Fe^2+^, MDA, GSH and GSSG in Caki-1 cells with vector/VDAC2 overexpression and DMSO/RSL3 (1μM) treatment. Data was normalized to the vector, DMSO group. **I-M.** Detection of cellular viability, Fe^2+^, MDA, GSH and GSSG in Caki-1 cells with vector/COL1A1 overexpression and DMSO/RSL3 (1μM) treatment. Data was normalized to the vector, DMSO group. Data are presented as mean ± SD, n = 3.

**
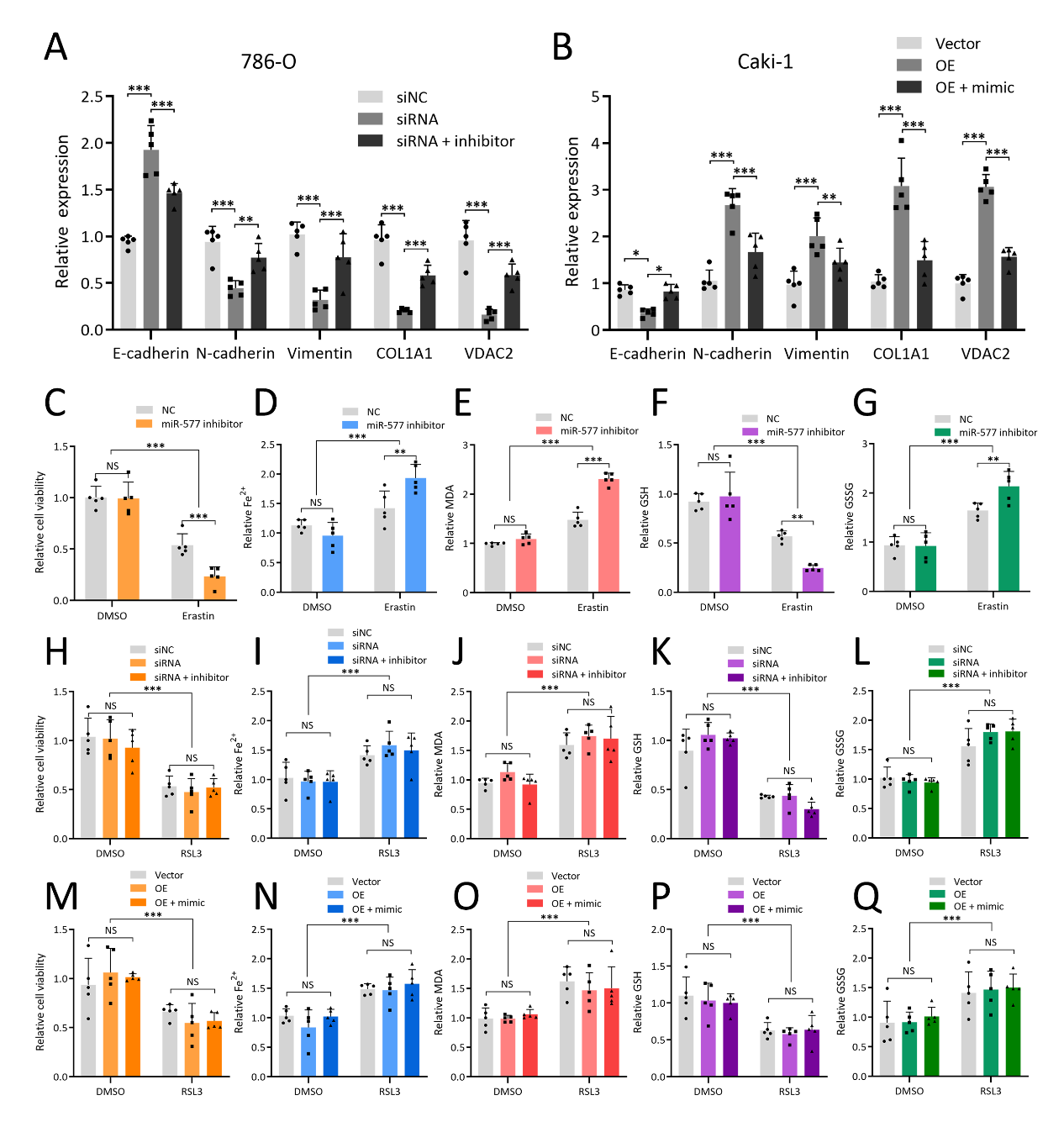
**

**Figure S7. Hsa_circ_0057105 balances EMT and ferroptosis through the miR-577/COL1A1/VDAC2 axis (supplementary information 2).** **A, B.** RNA levels of EMT markers (E-cad, N-cad and Vimentin), COL1A1 and VDAC2 in 786-O/Caki-1 cells rescue experiment. GAPDH was used as an internal control. **C-G.** Detection of cellular viability, Fe^2+^, MDA, GSH and GSSG in Caki-1 cells with NC mimic/miR-577 inhibitor and DMSO/erastin (5μM) treatment. Data was normalized to the NC mimic, DMSO group. **H-L.** Detection of cellular viability, Fe^2+^, MDA, GSH and GSSG in 786-O cells rescue experiment. Data was normalized to the siNC, DMSO group. 1μM RSL3 was used. **M-Q.** Detection of cellular viability, Fe^2+^, MDA, GSH and GSSG in Cak-1 cells rescue experiment. Data was normalized to the vector, DMSO group. 1μM RSL3 was used. Data are presented as mean ± SD, n = 3.


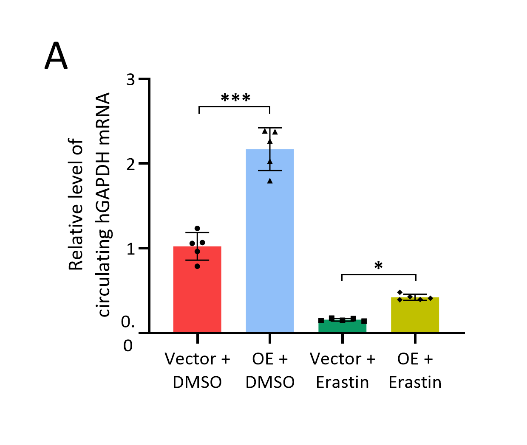


**Figure S8. Hsa_circ_0057105 balances EMT and ferroptosis through the miR-577/COL1A1/VDAC2 axis (supplementary information).** **A.** qRT-PCR analysis of human GAPDH mRNA in mouse circulation.

**Table S1 (please refer to Table S1.xlsx file)**

**Significantly-expressed circRNAs in the circRNA-seq.**

Detailed information of 212 differentially expressed circRNAs (>2-fold change, p <0.05) with circBase annotation.

**Table S2 (please refer to Table S2.xlsx file)**

**Correlation coefficients and p values of circRNAs and EMT ssGSEA score.**

List of correlation coefficients and p values of 29 circRNAs that are significant correlation with 8 EMT-related pathways.

**Table S****3 (please refer to Table S3.xlsx file)**

**Primers and DNA/RNA sequence used in this study.**

Primers and DNA/RNA sequence used in this study.
